# FBXO32-mediated degradation of PTEN promotes lung adenocarcinoma progression

**DOI:** 10.1038/s41419-024-06635-4

**Published:** 2024-04-20

**Authors:** Jie Wu, Ting Wen, Antonio Marzio, Dingli Song, Sisi Chen, Chengcheng Yang, Fengyu Zhao, Boxiang Zhang, Guang Zhao, Alessandra Ferri, Hao Cheng, Jiao Ma, Hong Ren, Qiao Yi Chen, Yiping Yang, Sida Qin

**Affiliations:** 1 Department of Thoracic Surgery, The First Affiliated Hospital of Xi’an Jiaotong University, Xi’an, Shaanxi China; 2 Department of Radiation Oncology, Shaanxi Provincial People’s Hospital, Xi’an, Shaanxi China; 3 Department of Cell Biology and Genetics, School of Basic Medical Sciences, Xi’an Jiaotong University, Xi’an, Shaanxi China; 4 Department of Pathology and Laboratory Medicine, Meyer Cancer Center, Weill Cornell Medical Center, New York, NY USA; 5 Department of Oncology, The Second Affiliated Hospital of Xi’an Jiaotong University, Xi’an, Shaanxi China; 6 Department of Oncology, The First Affiliated Hospital of Xi’an Jiaotong University, Xi’an, Shaanxi China; 7 Department of Rehabilitation, The First Affiliated Hospital of Xi’an Jiaotong University, Xi’an, Shaanxi China; 8 Clinical Research Center for Shaanxi Provincial Radiotherapy, Department of Radiation Oncology, Shaanxi Provincial Cancer Hospital, Xi’an, Shaanxi China; 9 Biobank, The First Affiliated Hospital of Xi’an Jiaotong University, Xi’an, Shaanxi China

**Keywords:** Ubiquitylation, Non-small-cell lung cancer

## Abstract

FBXO32, a member of the F-box protein family, is known to play both oncogenic and tumor-suppressive roles in different cancers. However, the functions and the molecular mechanisms regulated by FBXO32 in lung adenocarcinoma (LUAD) remain unclear. Here, we report that FBXO32 is overexpressed in LUAD compared with normal lung tissues, and high expression of FBXO32 correlates with poor prognosis in LUAD patients. Firstly, we observed with a series of functional experiments that FBXO32 alters the cell cycle and promotes the invasion and metastasis of LUAD cells. We further corroborate our findings using in vivo mouse models of metastasis and confirmed that FBXO32 positively regulates LUAD tumor metastasis. Using a proteomic-based approach combined with computational analyses, we found a positive correlation between FBXO32 and the PI3K/AKT/mTOR pathway, and identified PTEN as a FBXO32 interactor. More important, FBXO32 binds PTEN via its C-terminal substrate binding domain and we also validated PTEN as a bona fide FBXO32 substrate. Finally, we demonstrated that FBXO32 promotes EMT and regulates the cell cycle by targeting PTEN for proteasomal-dependent degradation. In summary, our study highlights the role of FBXO32 in promoting the PI3K/AKT/mTOR pathway via PTEN degradation, thereby fostering lung adenocarcinoma progression.

## Introduction

Lung cancer is the leading cause of cancer-related death worldwide [1]. The most common subtype of lung cancer is lung adenocarcinoma (LUAD), which accounts for ~40% of all lung cancer cases [2]. In the past decade, targeted therapy for LUAD has improved the prognosis of patients, but frequent target resistance prompts investigation into additional novel targets. F-box proteins participate in cancer-related signaling pathways by ubiquitinating and degrading targeted substrates [3, 4]. Among the F-box family members, FBXW7, SKP2 and β-TrCP have been meticulously studied, and their diagnostic and therapeutic applications are extensive in both preclinical and clinical studies [5–9]. However, the function and clinical value of other F-box proteins in LUAD require further exploration.

We analyzed the expression profile of F-box proteins in LUAD using TCGA datasets and found the top four upregulated F-box proteins in LUAD compared with normal lung tissues. FBXO32 was found to be one of the highest expressing genes and is positively correlated with poorer prognosis in LUAD patients. The prognosis of patients with LUAD is directly affected by invasive metastasis [10]. FBXO32 has been reported to play a vital role in tumor invasion and metastasis. In breast cancer, FBXO32 mediates CtBP1 ubiquitination and nuclear translocation to promote epithelial-to-mesenchymal transition (EMT) [11]. However, FBXO32 was reported to inhibit EMT by targeting the degradation of MyoD in bladder epithelial carcinoma [12]. Therefore, although FBXO32 has previously been reported to be involved in EMT in several types of tumors, its role in LUAD remains unclear.

In our study, we discovered that FBXO32 was significantly and highly expressed in LUAD and was positively associated with lymph node metastasis and poor patient prognosis. Furthermore, our findings indicated that FBXO32 regulates the cell cycle of LUAD cells and promotes EMT both in vivo and in vitro. Thus, we used a proteomic-based approach to investigate novel substrates of FBOX32 responsible for driving the aforementioned phenotypes. Among those, the tumor suppressor PTEN, with an established role in EMT, attracted our attention because it is one of the major players of the PI3K/AKT/mTOR pathway, and is also found to be correlated with FBXO32.

*PTEN* is tightly associated with tumor development, and the protein it encodes has been well documented to be either downregulated or functionally lost in several tumors, including LUAD [13–15]. The expression level of PTEN is regulated by ubiquitination and degradation [16, 17]. To validate PTEN as a novel potential substrate of FBXO32, we performed co-immunoprecipitation (Co-IP) and ubiquitination-related experiments, and detected the correlation between the expression of FBXO32 and PTEN in LUAD. In addition, double knock down of FBXO32 and PTEN further validated that FBXO32 promotes EMT and regulates cell cycle by degrading PTEN. Our study reveals that FBXO32 promotes lung adenocarcinoma progression by degrading PTEN via ubiquitination.

## Results

### Expression of FBXO32 is higher in LUAD and is associated with unfavorable prognosis

**Fig. 1:**
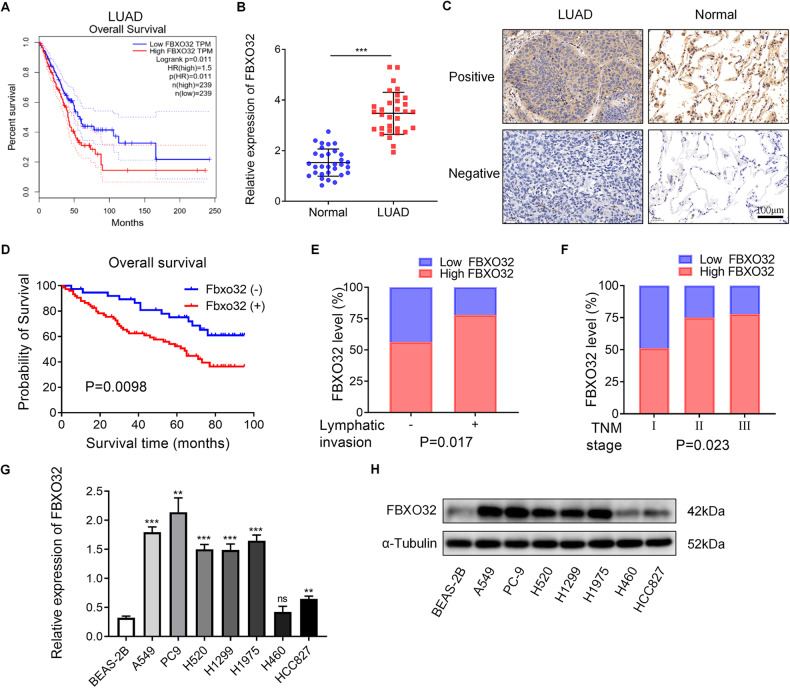
FBXO32 is overexpressed in LUAD. **A** The prognostic value of the FBXO32 expression level in LUAD from the GEPIA database. **B** The mRNA expression of FBXO32 in 30 pairs of LUAD tissues and paracancerous tissues. *P* values was determined by *t* test. **C** Representative immunohistochemistry images showing FBXO32 expression in normal lung tissues and LUAD tissues from patients with LUAD. **D** Survival curve of LUAD patients with high and low FBXO32 expression. *P* values were determined by log-rank test. **E**, **F** The proportion of patients with high- or low-FBXO32 in different lymphatic invasion stages (**E**) and TNM stages (**F**). *P* values were determined by chi-square tests. **G**, **H** mRNA and protein expression levels of FBXO32 in normal lung epithelial cells (BEAS-2B) and lung cancer cells (A549/PC9/H520/H1299/H1975/H460/HCC827) as measured by RT‒qPCR and western blotting analysis, respectively. Data were presented as means ± SEM. *P* values were determined by two-way ANOVA analysis.

To investigate the role of F-box family members in LUAD, we utilized RNA-Seq data from TCGA database and obtained a heatmap showing the expression profile of F-box genes in normal lung tissues and in LUAD tissues (Supplementary Fig. 1). Among these genes, *FBXO47*, *FBXO32*, *FBXO1* and *FBXO43* are the top upregulated F-box proteins in LUAD compared with normal lung tissues (Supplementary Fig. 2). Then, we analyzed the prognostic value of these four F-box proteins using the GEPIA database and found that high expression levels of *FBXO32* and *FBXO43* were significantly associated with unfavorable overall survival in LUAD (Fig. 1A and Supplementary Fig. 3). *FBXO43*, also named Emi2, is a well-established gene for regulating cell cycle in APCC, therefore we focused our attention on *FBXO32* in our study. Using the GEPIA database, we found that the expression of FBXO32 was significantly upregulated in both LUAD and LUSC samples compared with normal lung samples (Supplementary Fig. 4). However, a high level of FBXO32 was significantly associated with unfavorable overall survival in LUAD rather than LUSC (Fig. 1A and Supplementary Fig. 5). These results suggested that, between LUAD and LUSC, FBXO32 may play a more important role in LUAD.

Moreover, we validated the mRNA level of FBXO32 in 30 pairs of LUAD and paracancerous lung tissues by qPCR, as well as FBXO32 protein level in 112 LUAD tissues and 68 paracancerous lung tissues via immunohistochemistry (IHC). We found that FBXO32 mRNA was expressed at higher levels in LUAD tissues than in paracancerous lung tissues (Fig. 1B). Representative images of IHC analyses are shown in Fig. 1C. At the protein level, tumors from 74 (66.1%) patients showed high expression for FBXO32, and the paracancerous lung tissues from 25 (36.8%) patients showed high expression. The protein expression of FBXO32 in LUAD was significantly higher than that of the paracancerous lung tissues (Supplementary Table 1, *P* < 0.01). Additionally, we observed that patients with increased FBXO32 protein expression showed poorer prognosis (Fig. 1D). Univariate and multivariate analyses were performed to show that FBXO32 expression is an independent factor that can be used to evaluate prognosis in LUAD patients (Supplementary Tables 2 and 3). Furthermore, we found that patients with lymph node metastases or advanced lung cancer are more likely to display higher protein levels of FBXO32 (Fig. 1E, F and Table 1).

**Table 1:** Correlation between FBXO32 expression and clinicopathological characteristics.

	Total	Low	High	*χ*^2^	*P* value
Gender					
Male	53	17	36	0.154	0.695
Female	59	21	38		
Age					
≤65	68	22	46	0.192	0.662
>65	44	16	28		
Smoking					
Yes	47	14	33	0.62	0.431
No	65	24	41		
Differentiation					
High and middle	72	23	49	0.354	0.552
Low	40	15	25		
LN metastasis					
No	62	27	35	5.733	0.017
Yes	50	11	39		
Lobe					
Left	63	20	43	0.306	0.58
Right	49	18	31		
Location					
Central	20	7	13	0.012	0.911
Peripheral	92	31	61		
Tumor					
T1	28	13	15	3.543	0.315
T2	65	18	47		
T3	15	5	10		
T4	4	2	2		
TNM staging					
I	45	22	23	7.566	0.023
II	40	10	30		
III	27	6	21		

In addition, we measured FBXO32 expression levels in both normal lung epithelial cell line (BEAS-2B) and NSCLC cell lines (A549/PC9/H520/H1299/H1975/H460/HCC827) by RT-qPCR and western blotting, and found that the expression of FBXO32 in LUAD cells (especially in PC9 and A549 cells) was higher than that of BEAS-2B cells at both the mRNA and protein levels (Fig. 1G, H).

### FBXO32 induces the EMT and promotes cell migration and invasion both in vitro and in vivo

**Fig. 2:**
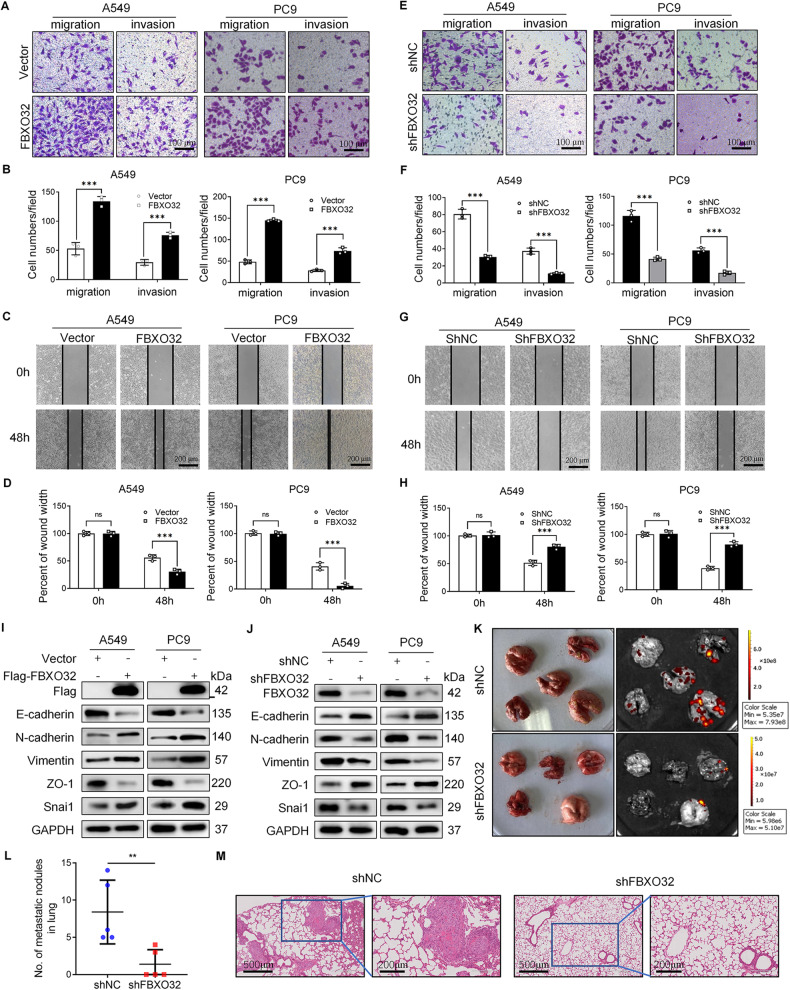
FBXO32 regulates the migration and invasion of LUAD cells. **A**–**D** Transwell assays and wound-healing assays in A549 and PC9 cells after FBXO32 overexpression. **E**–**H** Transwell assays and wound-healing assays in A549 and PC9 cells after FBXO32 knockdown. Migration and invasion cells per field were counted and analyzed (**B**, **F**). Percent of wound width were measured and analyzed at 0 h and 48 h after the wound formation (**D**, **H**). ***P < 0.001. ns no significance. *n* = 3. *P* values were determined by two-way ANOVA analysis. **I**, **J** The expression of cell migration- and invasion-related molecular markers after overexpression (**I**) or knockdown (**J**) of FBXO32 in A549 and PC9 cells as measured by western blotting. **K** Representative images showing the lung tissues of nude mice as imaged by a photo camera and bioluminescent imaging (BLI). A549-shNC or A549-shFBXO32 cells were injected through the tail vein of nude mice that were 4 weeks old. **L** The number of metastatic nodules were counted after the lung tissues of the nude mice were obtained. **P < 0.01, *n* = 5. *P* value was determined by *t* test. **M** H&E staining images of the lung tissues obtained from nude mice models of metastasis.

To analyze the function of FBXO32 in cell migration and invasion, we constructed FBXO32-overexpressing and FBXO32-knockdown LUAD cell lines. PC9 and A549 cells were transfected with wild-type FBXO32 plasmid to overexpress FBXO32. We used empty vector as control (Vector) and E3 ligase deficient FBXO32 plasmid as negative control (NC). Besides, PC9 and A549 cells transfected with sh-control or sh-FBXO32-1/sh-FBXO32-2 lentivirus were used as control group or FBXO32-knockdown group, respectively. Then, we performed transwell and wound-healing experiments and found that ectopic overexpression of wild-type FBXO32 enhanced the invasion and migration capacities compared to the control group (Fig. 2A–D and Supplementary Fig. 6). Moreover, knocking down FBXO32 significantly inhibited cell invasion and migration abilities of both A549 and PC9 cells. Interestingly, reintroducing wild-type, but not E3 ligase deficient FBXO32 in FBXO32-depleted cells can rescue the phenotypes. (Fig. 2E–H and Supplementary Fig. 7). Overexpression of FBXO32 promoted the reversion of epithelial-like morphological features, and therefore A549 cells presented a mesenchymal-like phenotype, which means that the junction between cells was decreased, and that the cells became slender and acquired a fibroblast-like mesenchymal phenotype (Supplementary Fig. 8). In addition, we evaluated the influence of FBXO32 on the expression of EMT-related markers via western blotting and immunofluorescence (IF). FBXO32 overexpression caused a decrease in E-cadherin and ZO-1 expression and an increase in the levels of N-cadherin, Vimentin, and Snail (Fig. 2I). Meanwhile, FBXO32 knockdown led to the opposite effect (Fig. 2J). Additionally, immunofluorescence results showed that, overexpressing FBXO32 reduced Vimentin (a mesenchymal cell marker) expression, and increased E-cadherin (an epithelial cell marker) expression. On the other hand, inverse expression patterns were observed after FBXO32 was knocked down (Supplementary Fig. 9). In summary, FBXO32 induces EMT and promotes cell migration and invasion in vitro.

To explore the influence of FBXO32 on LUAD metastasis in vivo, luciferase-positive A549 cells expressing either a non-targeting shRNA control (shNC) or shRNA against *FBXO32* (sh*FBXO32*) were intravenously injected into nude mice, and lung tissues were harvested after 4 weeks. Compared with that of the shNC group, the shFBXO32 group displayed lower number of lung metastatic nodules and decreased fluorescence intensity (Fig. 2K, L). In line with this result, H&E staining revealed that the number and size of lung metastatic nodules were reduced after *FBXO32* knockdown (Fig. 2M). Thus, our in vivo results corroborate the hypothesis that FBXO32 expression can lead to morphological transition and obtain a mesenchymal phenotype, resulting in the acquisition of migration and invasion properties in LUAD.

### FBXO32 alters cell cycle process and regulates the AKT/mTOR signaling pathway in LUAD

To further explore the function of FBXO32 in LUAD, we analyzed the RNA-seq data from TCGA database. Gene-set enrichment analysis (GSEA) displays the highly enriched biological process, cellular component, and molecular function terms obtained through Gene Ontology (GO) enrichment analysis. It is suggested that FBXO32 is highly involved in “extracellular matrix organization”, “extracellular structure organization” and “response to oxygen levels” in LUAD (Supplementary Fig. 10). The molecular function terms for FBXO32 were enriched in “cell adhesion molecule binding”, “extracellular matrix structure constituent”, and “integrin binding” and “growth factor binding”.

**Fig. 3:**
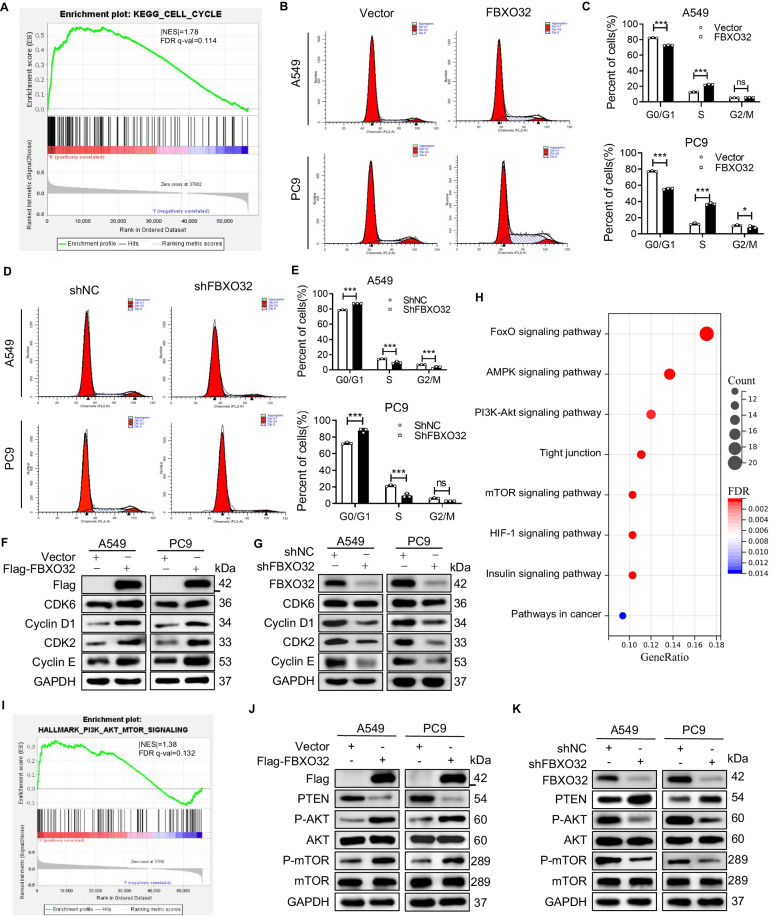
FBXO32 regulates the cell cycle and AKT/mTOR pathway in LUAD cells. **A** GSEA enrichment plot showing that FBXO32 is related to the cell cycle in LUAD. **B**–**E** FACS analysis showing cell cycle progression in A549 and PC9 cells after FBXO32 overexpression (**B**) or knockdown (**D**). Percentage of G0/G1 phase, S phase or G2/M phase cells were analyzed (**C**, **E**). ***P < 0.001; *P < 0.1; ns not significant. *P* values were determined by two-way ANOVA analysis**. F**, **G** Expression of cell cycle-related molecular markers after overexpression (**F**) or knockdown (**G**) of FBXO32 in A549 and PC9 cell lines as measured by western blotting. **H** Bubble plot showing FBXO32-related pathways identified by KEGG pathway analysis. **I** GSEA enrichment plots representing that FBXO32 is related to the PI3K/AKT/mTOR signaling pathway in LUAD. **J**, **K** Expression of the main markers of the AKT/mTOR signaling pathway in A549 and PC9 cells as measured by western blotting following the overexpression (**J**) or knockdown (**K**) of FBXO32.

To dive deeper into the underlying mechanisms of FBXO32 in LUAD, we performed GSEA using the KEGG database. We found that genes involved in the cell cycle were enriched in patients with high levels of FBXO32 (Fig. 3A). Then, we analyzed the cell cycle profile in FBXO32-overexpressing or FBXO32-knockdown A549 and PC9 cells, and found that fewer LUAD cells were blocked in the G1 phase and more cells entered the S phase when wild-type FBXO32 was stably overexpressed compared with empty vector (Vector) and E3 ligase deficient FBXO32 negative control (NC) groups (Fig. 3B, C and Supplementary Fig. 11). In contrast, more cells were arrested in the G1 phase, and fewer cells entered the S phase after knocking down FBXO32 by lentiviral transfection (Fig. 3D, E). Besides, reintroducing WT, but not E3 ligase deficient FBXO32 in FBXO32-depleted cells can rescue the phenotypes (Supplementary Fig. 12). In line with these results, we observed that ectopic expression of FBXO32 in A549 and PC9 cells significantly increased the expression of Cyclin D1, CDK2 and Cyclin E (Fig. 3F), while downregulation of FBXO32 by shRNA reduced the levels of Cyclin D1, CDK2 and Cyclin E (Fig. 3G).

Since FBXO32 alters cell cycle progression and promotes cell migration and invasion in LUAD, we performed GSEA using the RNA-seq data from TCGA database to further investigate gene signature pathways that are modulated by FBXO32. Our analysis suggested that FBXO32 levels are linked to the PI3K/AKT/mTOR signaling pathway (Fig. 3H). Besides, FBXO32 protein interaction network was analyzed by *STRING* (Supplementary Fig. 13), and we chose the top 200 proteins for KEGG pathway enrichment analysis using *DAVID* Bioinformatics Resources. We also found that FBXO32-related proteins are related to the PI3K/AKT/mTOR signaling pathway (Fig. 3I). To validate whether FBXO32 activated these pathways in LUAD, we measured the expression of the proteins related to these pathways by western blotting. The results showed that overexpression of FBXO32 downregulated the expression of PTEN and upregulated the expression of P-AKT and P-mTOR, but the expression of total AKT and total mTOR did not change (Fig. 3J). Consistently, the PI3K/AKT/mTOR signaling pathway was undoubtedly inactivated by knocking down FBXO32 (Fig. 3K).

### FBXO32 targets PTEN for ubiquitin‒proteasome degradation

To further identify the specific mechanism of FBXO32 involved in regulating LUAD, we carried out a proteomic screen to investigate the potential substrates interacting with FBXO32. PC9 cells transfected with either control or Flag-FBXO32 were treated with MG132 for 6 h, and then we harvested the proteins for IP-MS analysis. We performed SDS-PAGE gel electrophoresis and silver stain for the proteins to verify the reliability of the extracted IP proteins (Supplementary Fig. 14). We then investigated the potential substrate of FBXO32 by mass spectrum and found 89 proteins (bind only to Flag-FBXO32 but not to Flag) may interact with FBXO32. The top ten proteins are shown in Supplementary Fig. 15. Among the identified candidates, the tumor suppressor PTEN attracted our attention since it is involved in EMT regulation, it is related to the PI3K/AKT/mTOR signaling pathway, and we already observed that its level can be modulate by FBXO32 (Fig. 3J, K). Thus, we further validated PTEN as a potential substrate of FBXO32.

**Fig. 4:**
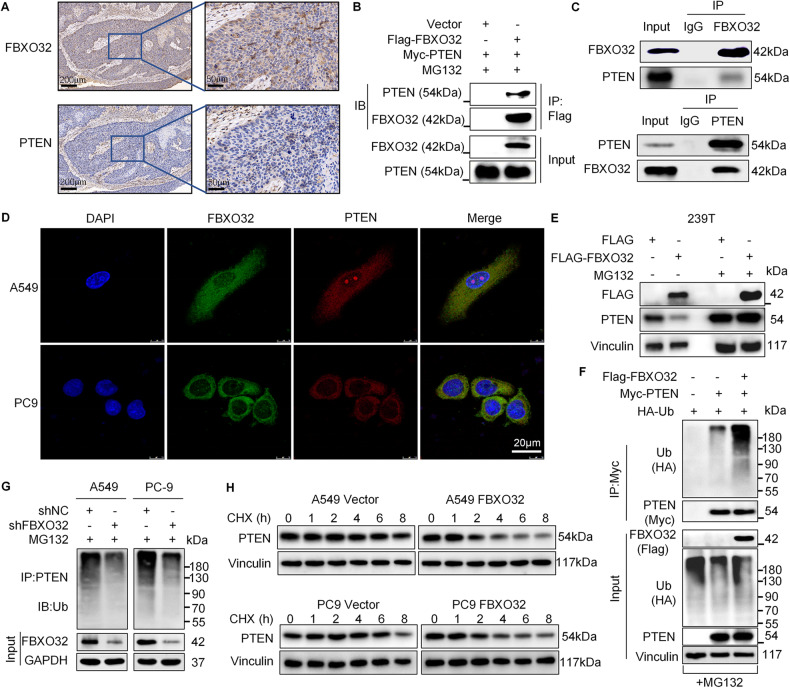
FBXO32 interacts with PTEN and mediates the ubiquitination of PTEN in LUAD. **A** Representative image of IHC showing the expression of PTEN and FXO32 in LUAD tissues. **B** Exogenous co-immunoprecipitation (co-IP) of FBXO32 and PTEN in 293T cells. **C** Endogenous co-IP with anti-FBXO32 or PTEN in the PC9 cell line followed by western blotting with anti-PTEN or anti-FBXO32 antibody. IgG was used as a negative control. **D** Immunofluorescence staining of FBXO32 (green) and PTEN (red) in LUAD cells; nuclear DNA was counterstained with DAPI (blue). **E** Expression of PTEN in transfected 293T cells with or without proteasome inhibitor MG132 treatment (10 μM for 6 h) as measured by western blotting. **F** Ubiquitinated PTEN in HEK293T cells co-transfected with the indicated plasmids as measured through a ubiquitination assay. **G** The results of the ubiquitination assay showing ubiquitinated PTEN in FBXO32-knockdown A549 and PC9 cells. **H** A549 and PC9 cells were treated with CHX (50 μg/ml) for the indicated times after FBXO32 overexpression. The protein expression of PTEN was examined by western blotting.

Firstly, we analyzed the expression of FBXO32 and PTEN in 112 LUAD samples through IHC and discovered that the expression of FBXO32 was negatively correlated with PTEN (Supplementary Table 4, *r* = −0.413, *P* < 0.001). Representative images of IHC are shown in Fig. 4A. Then, we found that PTEN has lower expression in lung adenocarcinoma cells comparing with normal bronchial epithelial cells (BEAS-2B) (Supplementary Fig. 16). To further analyze the relationship between FBXO32 and PTEN, we performed exogenous and endogenous co-IP experiments to verify that FBXO32 and PTEN interact with each other (Fig. 4B, C). By immunofluorescence staining, we observed that FBXO32 and PTEN colocalized in the cytoplasm of A549 and PC9 cells (Fig. 4D). Besides, we extracted cytoplasmic protein and cell membrane protein of lung adenocarcinoma cells respectively and found that FBXO32 and PTEN were co-expressed in the cytoplasm rather than in the cell membrane. Then, we examined the binding of FBXO32 and PTEN in cytoplasmic protein through co-immunoprecipitation and detected their binding (Supplementary Fig. 17). To further investigate the regulatory relationship between FBXO32 and PTEN, we knocked down FBXO32 and found that the expression of PTEN was upregulated, but when we knocked down PTEN, the expression of FBXO32 was not significantly changed (Supplementary Fig. 18). This finding indicated that FBXO32 acted as an upstream regulator to influence the expression of PTEN. Furthermore, PTEN was degraded by wild-type FBXO32, but not E3 ligase deficient FBXO32, and treatment with the proteasome inhibitor MG132 blocked the degradation of PTEN caused by FBXO32 (Fig. 4E and Supplementary Fig. 19). In addition, we found that knocking down FBXO32 exerted no significant effect on the mRNA level of PTEN, which suggested that FBXO32 regulated PTEN through post-translational modification (Supplementary Fig. 20). Next, we performed a ubiquitin assay with 293T cells transfected with HA-Ub, Myc-PTEN, Flag-FBXO32 or empty plasmid, and found that FBXO32 overexpression markedly increased PTEN ubiquitination (Fig. 4F). We also performed an ubiquitin assay with A549 and PC9 cells transfected with either shNC or sh*FBXO32* and found that knocking down FBXO32 notably decreased ubiquitinated PTEN (Fig. 4G). In line with this result, the stability of PTEN was decreased after FBXO32 overexpression (Fig. 4H). Altogether, the above results demonstrate that FBXO32 controls the ubiquitin-mediated degradation of PTEN.

**Fig. 5:**
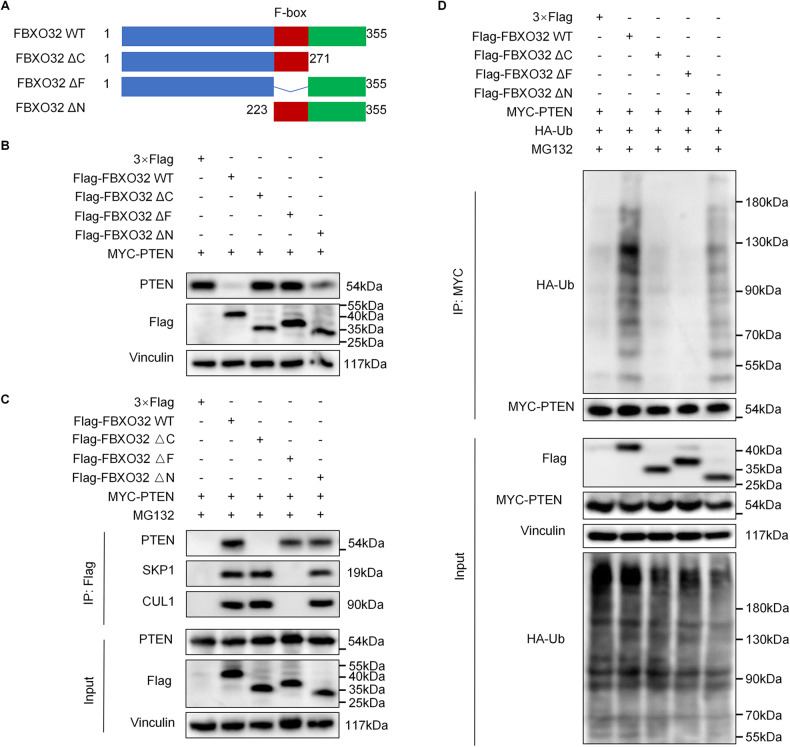
FBXO32 recognizes and interacts with PTEN through its C-terminus. **A** Wild-type FBXO32 and FBXO32 deletion mutants schematics. **B** Effect of FBXO32 deletion mutants on PTEN degradation in 293T cells as determined by western blotting. **C** 293T cells transfected with Myc-tagged PTEN and Flag-tagged wild-type FBXO32 or FBXO32 deletion mutants (FBXO32 ΔC, FBXO32 ΔF or FBXO32 ΔN) were treated with MG132 (10 μM) for 6 h and then harvested for immunoprecipitation and immunoblotting. **D** HEK293T cells were transfected with Myc-PTEN, HA-Ub and FLAG-FBXO32 WT or FBXO32 deletion mutants and incubated with MG132 after 24 h. The ubiquitination of PTEN was analysed.

FBXO32 consists of an N-terminal domain, an F-box domain and a C-terminal domain. To further study which domain mediates the regulation of PTEN by FBXO32, we constructed an FBXO32 wild-type plasmid and specific FBXO32 domain-deleted plasmids (Fig. 5A). After transfecting 293T cells with the indicated plasmids, we first investigated the influence of wild-type or mutant FBXO32 on PTEN degradation through western blotting and found that PTEN was not degraded by FBXO32 mutants lacking either the F-box domain or C-terminus (Fig. 5B). Next, to further explore the domain through which FBXO32 binds to PTEN, we detected the binding of wild-type or specific domain-deficient FBXO32 with PTEN, SKP1, and CUL1 by co-immunoprecipitation. We found that FBXO32 lacking F-box domain could bind to PTEN, but could not bind to SKP1 and CUL1. FBXO32 without C-terminal domain could bind to SKP1 and CUL1, but not PTEN. FBXO32 without N-terminal domain can still normally bind to PTEN, SKP1 and CUL1 (Fig. 5C). These results indicated that the F-box domain of FBXO32 is responsible for its binding to SKP1 and CUL1, and the C-terminal domain mediates the binding of FBXO32 to PTEN. Additionally, we examined the effect of FBXO32 lacking different domains on PTEN ubiquitination by ubiquitination assays. As shown in Fig. 5D, FBXO32 lacking the N-terminal domain barely affected the ubiquitination of PTEN, while FBXO32 lacking either the F-box domain or the C-terminal domain could barely ubiquitinate PTEN. Taken together, FBXO32 binds to PTEN through its C-terminal domain and binds to SKP1 and CUL1 through its F-box domain, both of which are essential for FBXO32-mediated PTEN ubiquitination and degradation.

### PTEN reverses the oncogenic effects of FBXO32 on LUAD both in vitro and in vivo

**Fig. 6:**
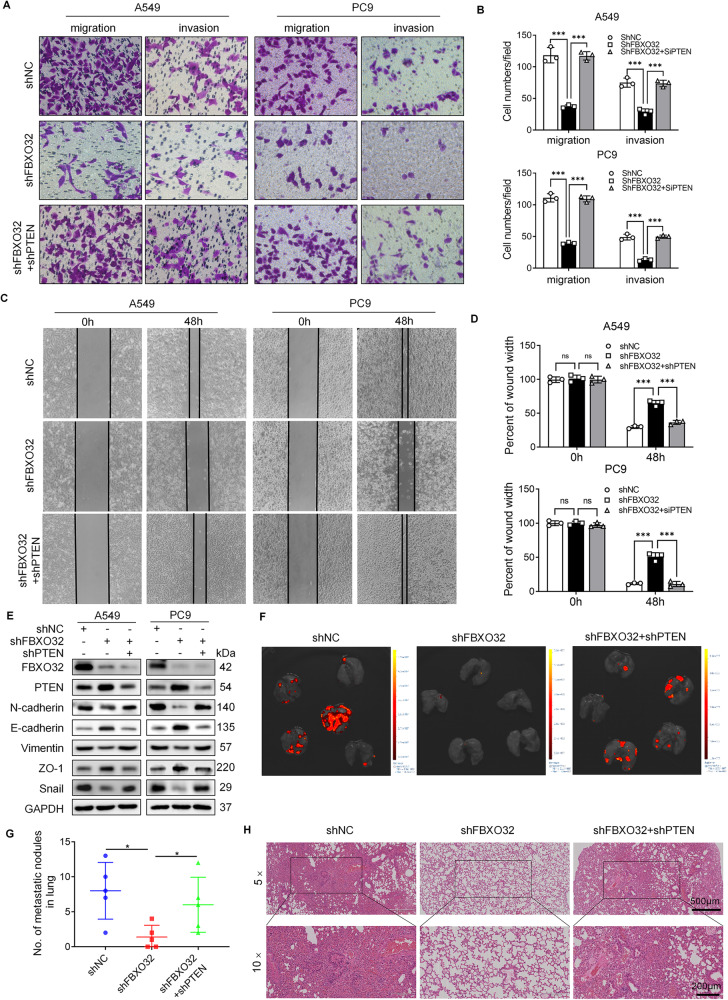
FBXO32 facilitates the migration and invasion of LUAD cells by downregulating PTEN. **A**–**D** The ability of cancer cells to migrate and invade was assessed using the transwell assay (**A**, **B**) and the wound-healing assay (**C**, **D**). Migration and invasion cells per field were counted (**B**). Percent of wound width were measured at 0 h and 48 h after the wound formation (**D**). ***P < 0.001. ns no significance. *P* values were determined by two-way ANOVA analysis. **E** The expression of cell migration- and invasion-related molecular markers after FBXO32 stable knockdown and FBXO32/PTEN double knockdown in A549 and PC9 cells was examined by western blotting. **F** Representative images of the lung tissues of nude mice as imaged by bioluminescent imaging (BLI). The nude mice were injected with A549-shNC, A549-shFBXO32 or A549-shFBXO32+shPTEN cells in the tail vein when 4 weeks old. **G** The number of metastatic nodules were counted after the lung tissue of nude mice was obtained. *P < 0.05. *P* values were determined by one-way ANOVA analysis. **H** HE staining images of lung tissues obtained from nude mouse metastasis models.

Based on previous results, we hypothesized that FBXO32 mediates PTEN ubiquitination and degradation to promote cell invasion and metastasis in LUAD. To this end, we downregulated the expression of PTEN in FBXO32-deficient A549 and PC9 cells. Knocking down *PTEN* abrogated the suppression of A549 and PC9 cell migration and invasion induced by *FBXO32* knockdown, as determined via transwell and wound-healing assays (Fig. 6A–D). In line with this result, *PTEN* co-knockdown reduced the upregulation in E-cadherin and ZO-1 expression that had been induced by *FBXO32* deficiency and increased the extent to which N-cadherin, Vimentin, and Snail expression was downregulated by *FBXO32* deficiency in A549 and PC9 cells (Fig. 6E). In addition, we found that PI3K inhibitor could reverse the EMT phenotype driven by FBXO32 overexpression, which indicated that FBXO32 promotes EMT phenotypes through activating PI3K/AKT/mTOR pathway (supplementary Fig. 21). We next examined whether the results obtained with cell systems could be recapitulated at the organismal level. A549 cells stably transfected with luciferase-tagged non-targeting control, *FBXO32*, or both *FBXO32* and *PTEN* shRNAs were injected intravenously into immunodeficient nude mice. The number and fluorescence intensity of the lung metastatic nodules were notably greater and much higher in the sh*FBXO32*+sh*PTEN* group than in the sh*FBXO32* group. Moreover, the nodules in the sh*FBXO32*+sh*PTEN* group were similar to those in the control group (Fig. 6F, G). Representative H&E staining images showing lung tissues from different groups are presented in Fig. 6H. These results suggested that the co-knockdown of *PTEN* reversed the suppression of tumor metastasis caused by *FBXO32* deficiency both in vitro and in vivo.

**Fig. 7:**
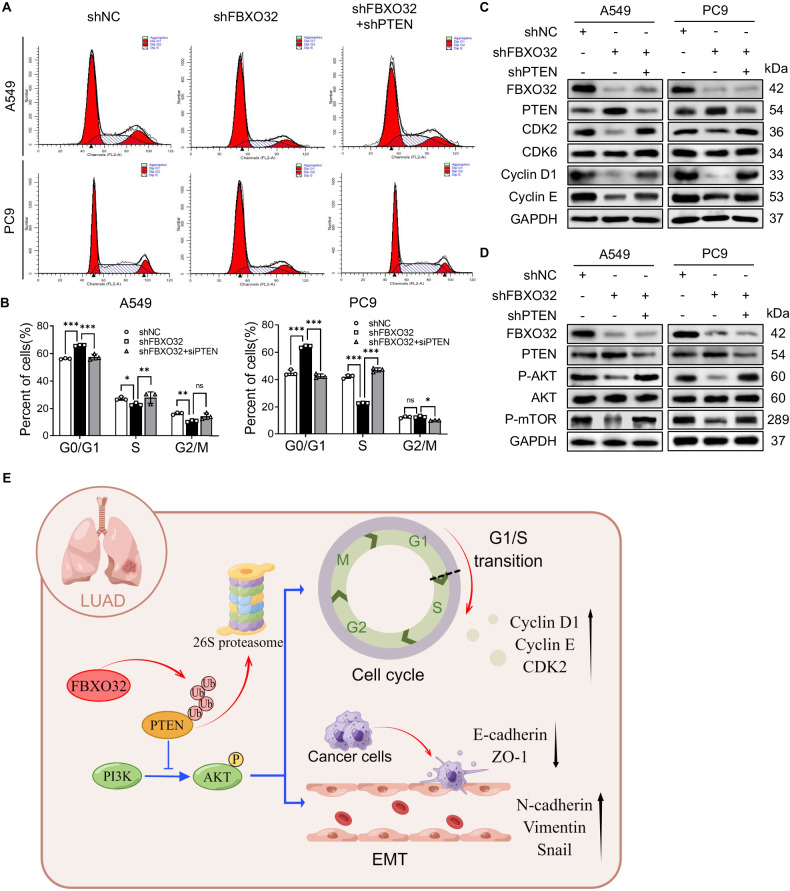
FBXO32 activates AKT/mTOR pathway and promotes cell cycle process by downregulating PTEN in LUAD. **A**, **B** A549 and PC9 cells with stable NC or FBXO32 knockdown and FBXO32/PTEN double knockdown were established. Cell cycle progression was analyzed by FACS. **P < 0.01. *P < 0.1. *P* values were determined by two-way ANOVA analysis. **C** The expression of cell cycle-related molecular markers as measured by western blot analysis. **D** The expression of FBXO32 and the main marker of the AKT/mTOR signalling pathway in A549 and PC9 cells was determined by western blotting. **E** Schematic diagram showing the regulation mechanism of FBXO32 in LUAD.

In order to validate that FBXO32 alters the cell cycle process in a PTEN-dependent manner. We detected cell cycle and the expression of G1-S phase-related proteins in control, *FBXO32* knockdown, and *PTEN*/*FBXO32* co-depletion A549 and PC9 cells. Interestingly, we observed that *PTEN*/*FBXO32* co-knockdown relieved G1 phase arrest caused by *FBXO32* knockdown and promoted cells entry into the S phase (Fig. 7A, B). Besides, the downregulated expression of G1-S phase-related proteins (Cyclin D1, CDK2 and Cyclin E) by *FBXO32* knockdown was reversed by knocking down *PTEN* (Fig. 7C). In addition, we also measured the expression of the proteins related to the PI3K/AKT/mTOR signaling pathway and verified that knocking down *PTEN* rescued the inhibition of the AKT/mTOR signaling pathway in *FBXO32*-depleted LUAD cells (Fig. 7D). Taken together, our results indicate that downregulation of *PTEN* (a) restores the AKT/mTOR pathway activation that had been inactivated by *FBXO32* knockdown; and (b) mitigates the suppression of cell cycle progression, and hinders cell migration and invasion induced by *FBXO32* deficiency.

Altogether, our results demonstrated that FBXO32 promotes PTEN degradation to activate PI3K/AKT/mTOR signaling and to induce cell migration and invasion in LUAD. Furthermore, FBXO32 also induces the G1–S phase transition via upregulated Cyclin D1, CDK2 and Cyclin E in a PTEN-dependent manner (Fig. 7E).

## Discussion

F-box proteins are crucial for cancer development, diagnosis and treatment. FBXW7, SKP2 and β-TrCP are the most intensively studied F-box proteins. However, the role of other F-box proteins in tumors needs to be further explored. We found that FBXO32 was one of the most significantly differentially expressed F-box proteins between LUAD tissues and normal lung tissues by analyzing RNA-Seq data from the TCGA database, which deserves further exploration.

At present, the role played by FBXO32 in tumorigenesis and progression is debated, and it has been shown that FBXO32 can act as either a tumor-promoting or a tumor-suppressing gene. Sahu et al. showed that FBXO32 mediates K63 ubiquitination of CtBP1 to promote EMT in breast cancer [11], while Tanaka et al. found that FBXO32 inhibited the EMT in bladder epithelial carcinoma by targeting the degradation of MyoD [12]. In our study, we discovered that FBXO32 promoted the invasion and metastasis of LUAD in vivo and in vitro. Also, FBXO32 promoted G1/S transition in LUAD. In our clinical data, we found that higher FBXO32 expression represents poorer prognosis with more lymph node metastases in LUAD patients. These results established the significance of FBXO32 as a driver gene in the progression of LUAD, which suggest that lung adenocarcinoma patients with higher expression of FBXO32 exhibit faster tumor progression, are more likely to develop distant metastasis, and experience poorer prognosis. Thereby, targeting FBXO32 is a promising potential therapeutic strategy to inhibit the progression of lung adenocarcinoma and improve the prognosis of patients. In our follow-up study, we plan to design a small molecule inhibitor of FBXO32 and construct an in vivo model in nude mice to investigate whether the small molecule inhibitor of FBXO32 can inhibit the progression of lung adenocarcinoma, in order to enhance the persuasiveness of FBXO32 as a feasible therapeutic target for lung adenocarcinoma.

To explore the underlying regulatory mechanism of FBXO32 in LUAD, we used TCGA database to perform bioinformatics analyses and revealed that FBXO32 was closely related to the PI3K/AKT/mTOR pathway. Moreover, we used a proteomic-based approach to investigate novel substrates of FBOX32 and discovered PTEN. Next, additional experiments were performed to validate PTEN as a novel potential substrate of FBXO32. First of all, we found that the expression of FBXO32 was negatively correlated with PTEN in LUAD patient samples. Next, we proved that FBXO32 targets PTEN for ubiquitin‒proteasome degradation. Previous studies indicated that the degradation of PTEN was also regulated by other E3 ubiquitin ligases, such as NEDD4 and WWP2, which indicated that FBXO32 may act synergistically with other E3 ubiquitin ligases to regulate PTEN expression in LUAD. Nonetheless, it is worth exploring the mechanisms of FBXO32-targeted degradation of PTEN.

FBXO32, a member of the F-box family, consists of an N-terminal domain, an F-box domain and a C-terminal domain. Studies have shown that the structural domains of FBXO32 recognize different substrates. Zhou et al. found that the C-terminal (228–355 aa) of FBXO32 bound to KLF4 for ubiquitination and degradation [18]. In contrast, Wu et al. found that FBXO32 bound to p21 via its N-terminus (1–50 aa), independent of its C-terminus or F-box domain [19]. How does FBXO32 interact with PTEN in LUAD? In our study, we revealed that FBXO32 recognized and bound to PTEN specifically via its C-terminal domain and the F-box domain helped FBXO32 bind to SKP1 and CUL1 to form SCF complexes in order to degrade PTEN.

PTEN is involved in regulating multiple cellular processes, such as cell cycle, migration, proliferation, and energy metabolism [13, 20]. It mainly acts as an upstream suppressor of the PI3K/AKT/mTOR signaling pathway [21]. It was reported that reduction of PTEN drives EMT and promotes migration and invasion in nasopharyngeal carcinoma and prostate cancer [22, 23]. In addition, previous studies reported that PTEN inhibits the G1/S transition in the cell cycle [24–28]. According to our findings, FBXO32 induced EMT and promoted the cell cycle G1/S transition in LUAD. Besides, we also found that FBXO32 targeted PTEN for ubiquitin-proteasomal degradation and activated the AKT signaling pathway. Based on these, we speculate that FBXO32 degrades PTEN through ubiquitination and activates the AKT signaling pathway, thereby promoting EMT and G1/S transition in LUAD. Therefore, we performed a series of rescue experiments and demonstrated that FBXO32 alters the cell cycle process and promotes the metastasis of LUAD in a PTEN-dependent manner. The utilization of PI3K/AKT/mTOR inhibitors could enhance progression-free survival (PFS) among individuals with tumors, but treatment-related adverse reactions often lead to forced drug discontinuation [29–31]. Previous studies have found that combination of drugs can reduce the occurrence of adverse drug reactions [32–34]. In the future, the combination of FBXO32 inhibitors with PI3K/AKT/mTOR inhibitor may be a potential way to reduce drug resistance and toxicity in lung adenocarcinoma.

In summary, we found that FBXO32 targeted PTEN for degradation through ubiquitination, thereby relieving the inhibitory effect of PTEN on the AKT/mTOR pathway, inducing EMT and promoting cell migration and invasion. FBXO32 also induces the G1/S phase transition in a PTEN-dependent manner in LUAD. As an important upstream regulator of the powerful tumor suppressor PTEN, FBXO32 is anticipated to be a new target for the diagnosis and treatment of LUAD.

## Methods

### Cell culture

NSCLC cells (A549, H1975, HCC827, PC-9, H1299 and H460), bronchial epithelial cells (BEAS-2B), and human embryonic kidney 293T cells were obtained from the ATCC. A549, HCC827, BEAS-2B and HEK293T cells were cultured in DMEM (HyClone, USA) with 10% fetal bovine serum (FBS) (Gibco, USA). H1975, H1299, H460 and PC-9 cells were cultured in RPMI-1640 medium (HyClone, USA) with 10% FBS (Gibco, USA). All cells were maintained at 37 °C in an incubator with 5% CO_2_. All cells were authenticated by STR profiling and tested for mycoplasma contamination.

### LUAD patient samples

All specimens were collected from 112 LUAD patients (IA-IIIB; 59 females and 53 males; age range: 39–80 years) who underwent pulmonary surgery between November 2014 and October 2019 at the First Affiliated Hospital of Xi’an Jiaotong University (Xi’an, China). Clinicopathological data, including gender, age, histology, tumor differentiation, tumor location, tumor size, lymph node metastasis, pTNM stage and overall survival, were collected for all cases. All patients had a single tumor without distant metastasis, and none of them had previously been treated with chemo- or radiotherapy. The study was approved by the Ethics Committee of the First Affiliated Hospital of Xi’an Jiaotong University and informed consent was obtained from all subjects. The clinical features of these samples are presented in Table 1.

### Cell transfection

Cell transfection in this study mainly involved lentiviral shRNA transfection and plasmid transfection. All cell transfections were performed using Lipo8000 transfection reagent (Beyotime, China) according to the manufacturer’s protocol. The lentiviral shRNAs (Hanbio, China) used in this study are listed in Supplementary Table 5.

### Western blotting analysis

Following the indicated treatments, RIPA buffer (Thermo Fisher Scientific, USA) was used to extract the total protein or nuclear protein from cells. The total protein was sampled and electrophoretically separated in 6-12% SDS-polyacrylamide gel (NCM Biotech, China) before being wet-transferred onto a membrane. After blocking with 5% skim milk powder (Beyotime, China) for 1 h, primary antibodies were incubated with the membrane at 4 °C overnight. Tris-buffered saline with Tween 20 (Beyotime, China) was used to wash the membrane three times for 10 min each time. Then, membranes were incubated with secondary antibodies for 2 h at room temperature. To visualize protein expression, the Hypersensitive Enhanced Chemiluminescence kit (Fdbio Science, China) was used. Antibodies used in this research are as follows. Primary antibodies: FBXO32 (abcam, Cat# ab168372), FBXO32(MAFbx) (Santa Cruz Biotechnology,Cat# sc-166806), VINCULIN (Santa Cruz Biotechnology, Cat# sc-73614), Akt (pan) (Cell Signaling Technology, Cat# 4685), Phospho-Akt (Ser 473) (Cell Signaling Technology, Cat# 4060), PTEN (Cell Signaling Technology, Cat# 9559), GAPDH (Proteintech, Cat#60004-1-Ig), Ubiquitin (Santa Cruz Biotechnology, Cat# sc-8017), Phospho-mTOR (Ser2448) (Cell Signaling Technology Cat# 5536), c-MYC (Proteintech, Cat# 10828-1-AP), Cyclin D1 (Proteintech, Cat# 60186-1-Ig), Cyclin E (Santa Cruz Biotechnology, Cat# sc-247), CDK6(Santa Cruz Biotechnology, Cat# sc-7961), CDK2 (Santa Cruz Biotechnology, Cat# sc-6248), E-Cadherin (Cell Signaling Technology, Cat# 14472), N-Cadherin (Cell Signaling Technology, Cat# 5741), Vimentin (Cell Signaling Technology, Cat# 13116), ZO-1 (Cell Signaling Technology, Cat# 13663), Slug (Cell Signaling Technology, Cat# 9585), Snail (Cell Signaling Technology, Cat# 3879), Lamin-B1 (abcam, Cat# ab133741), HA (Santa Cruz Biotechnology, Cat# sc-7392), FLAG (Beyotime, Cat# AF519), CUL1 (abcam, Cat# ab75817), SKP1 (Cell Signaling Technology, Cat# 2156), β-tubulin (Proteintech, Cat# 10094-1-AP), MYC (abcam, Cat# ab32). Secondary antibodies: Anti-mouse IgG, HRP-linked Antibody(Cell Signaling Technology, Cat# 7076), Anti-rabbit IgG, HRP-linked Antibody (Cell Signaling Technology, Cat# 7074).

### Reverse transcriptase-polymerase chain reaction (RT‒PCR)

Total RNA was extracted from cells and tissues using a Fast 2000 kit (Fastagen, China) following the manufacturer’s protocol. cDNA was synthesized using a Reverse Transcription Kit (CWBIO, China), followed by real-time PCR using a SYBR Green qPCR Kit (CWBIO, China) and CFX96 Real-time PCR detection system (Bio-Rad). The 2-ΔΔCt method was used to compute the fold difference. The primers used in this study are presented in Supplementary Table 6.

### Immunohistochemistry (IHC)

Primary human LUAD samples were collected for immunohistochemistry staining to measure protein expression. Immunohistochemistry was performed according to a previous method [35]. The antibodies used for IHC in this study are as follows. FBXO32 (Proteintech, Cat# 67172-1-Ig, 1:500), PTEN (Proteintech, Cat# 22034-1-AP, 1:500).

### Cell cycle analysis

Cell cycle analysis was performed using flow cytometry [36]. The specific operational steps were carried out in accordance with a cell cycle analysis kit’s instruction. (Yeasen Biotechnology, 40301ES). Briefly, A549 and PC9 cells were collected by trypsinization and washed with PBS after transfection with the indicated shRNA or plasmid after culturing for 24 h. Next, the cells were fixed with cold 70% ethanol overnight at 4 °C, followed by washing twice with PBS. The cells were then resuspended in a mixture of PI and RNAase solution and analyzed by flow cytometry. The data were processed with FlowJo software (FlowJo, RRID:SCR_008520).

### Transwell migration and invasion assays

In all, 8.0-μm 24-Transwell permeable supports (Corning, USA) were used for Transwell assays. The chambers used in the Transwell invasion assay were precoated with Matrigel matrix (BD Biosciences, USA). A total of 2 × 10^4^ cells were starved after 24 h, diluted in 200 µl of serum-free medium and seeded into upper Transwell chambers, and 600 μL of medium containing 10% FBS was added to each of the lower chamber. After 24–48 h, the chambers were removed, and the cells in the chamber were wiped off with cotton swabs. Next, the cells on the bottom surface of the membrane were fixed with methanol solution for 10 min and dyed with 0.5% crystal violet solution (Beyotime, China) for 20 min. The migrating and invading cells were photographed and counted under a Nikon inverted microscope.

### Immunofluorescence staining

After the indicated transfection, the cells were fixed with 95% ethanol for 20 min and permeabilized for 10 min on ice with 0.2% Triton X-100 (Sigma-Aldrich, China). Next, the cells were blocked with 5% bovine serum albumin (BSA) for 1 h at room temperature. The cells were then treated with the corresponding primary antibodies at 4 °C overnight, followed by fluorochrome-conjugated secondary antibodies. DAPI (Sigma, China) was utilized for DNA staining, and the cells were then sealed after 30 min. Finally, image capture was performed using confocal laser microscopy (Leica, Germany) or inverted fluorescence microscopy (Leica, Germany). All antibodies used in this study are as follows. Primary antibodies: E-Cadherin (Cell Signaling Technology, Cat# 14472, 1:200), Vimentin (Cell Signaling Technology, Cat# 13116, 1:200), FBXO32 (abcam, Cat# ab168372, 1:200). Secondary antibodies for IF: anti-Rabbit IgG FITC-conjugated secondary antibody (Proteintech, Cat# SA00003-2, 1:200), anti-Rabbit IgG CY3-conjugated secondary antibody (Proteintech, Cat# SA00009-2, 1:100).

### In vivo mouse model

BALB/c nude mice were used to construct the metastasis model. 4-week-old nude mice, half male and half female, with similar body weight were randomly divided into experimental and control groups. For animal studies, the sample size was estimated from previous studies and each group contains 5–6 mice. A549 cells stably expressing luciferase-tagged shNC, shFBXO32, shFBXO32, or shPTEN were established. To construct the metastatic tumor model, 200 µl of PBS containing 1 × 10^6^ cells was injected into the tail vein of nude mice. Necropsy followed by bioluminescent imaging (BLI) were performed to identify metastases after 4 weeks. The lung tissues were collected for HE staining. Mice were bred in the Animal Research Center of Xi’an Jiaotong University. The Guidelines for the Care and Use of Laboratory Animals of the National Institute of Health in China were followed in terms of both animal care and experimentation. All animal experiments were approved by the Ethics Committee of the First Affiliated Hospital of Xi’an Jiaotong University.

### Immunoprecipitation followed by mass spectrometry (IP-MS)

PC9 cells were transfected with Flag-FBXO32 or Flag-Control plasmids. After 24 h, the IP proteins were harvested with IP buffer and anti-Flag antibody (Beyotime, Cat# AF519) and then subjected to MS analysis. The two groups of proteins were electrophoresed in SDS-PAGE gels, and when the proteins entered the lower gel about 2 cm, the gel strip containing the proteins was cut off from the whole gel and placed in EP tubes containing ddH2O. The gel strips prepared as described above were sent to Jingjie PTM Biolabs (Hangzhou, China) for mass spectrometry detection. Firstly, the proteins in the gel were digested into peptides and separated from the gel, then the peptides were detected by liquid chromatography and mass spectrometry, and finally the specific information of the peptides was determined by data searching. The proteins and peptides identified by the Flag control group and the FBXO32 experimental group as being able to bind respectively were integrated and analyzed, and the proteins and peptides within the intersection of the two groups were removed, and then the proteins and peptides that could specifically bind to FBXO32 were screened according to the scores.

### Co-immunoprecipitation (Co-IP)

For endogenous immunoprecipitation, cells were treated with MG132 (20 µM) for 6 h before being lysed with RIPA buffer. The indicated antibodies and cell lysates were incubated at room temperature for 2 h, and then Protein A/G PLUS-Agarose (Santa Cruz Biotechnology, USA) was incubated with the lysate overnight at 4 °C. The beads were washed three times with RIPA buffer and analyzed by immunoblot analysis. For exogenous immunoprecipitation, HEK293T cells were transfected with the indicated plasmids to exogenously overexpress specific proteins. The next steps were performed following the same steps used for the endogenous immunoprecipitation experiments. The antibodies used for the IP experiments are FLAG (Beyotime, Cat# AF519), FBXO32(MAFbx) (Santa Cruz Biotechnology, Cat# sc-166806), PTEN (Cell Signaling Technology, Cat# 9559), and MYC (abcam, Cat# ab32).

### In vivo ubiquitination assay

Ubiquitination assays were carried out as reported previously [37]. ShNC or ShFBXO32 was transfected into A549 and PC9 cells. Plasmids encoding the proteins HA-Ub, FLAG-FBXO32, MYC-PTEN, and other indicated proteins were transfected into HEK293T cells. Before being lysed with RIPA buffer, the cells were treated with 20 µM of MG132 for 6 h. As stated in the Co-IP experimental method section, anti-PTEN or anti-MYC-conjugated agarose beads were used to co-immunoprecipitate the lysate. Western blotting was performed to measure ubiquitinated proteins using an anti-ubiquitin antibody (Santa Cruz Biotechnology Cat# sc-8017, RRID: AB_628423).

### Bioinformatic analysis

RNA-Seq data and related clinical data of TCGA LUAD and LUSC samples were obtained from Genomic Data Commons. Expression heatmap of F-box family members in LUAD was drawn by using the “heatmap” package in R software. Survival analysis was performed using the Kaplan‒Meier Plotter database and GEPIA database. Differential gene expression analysis and correlation analysis were also performed using the online database GEPIA. Gene set enrichment analysis (GSEA) (Gene Set Enrichment Analysis, RRID:SCR_003199) was performed to determine the function of FBXO32. R software was used for Gene Ontology (GO) term enrichment analysis. The 200 most promising proteins interacting with FBXO32 were obtained from the STRING (STRING, RRID:SCR_005223) database and then placed into the DAVID database for further KEGG analysis. The online links of all the databases used in our study are listed in Supplementary Table 7.

### Statistics

Statistical analyses were performed using GraphPad Prism 8 (GraphPad Prism, RRID:SCR_002798). All in vitro experiments were performed in triplicates or a higher replicate unless otherwise stated. Most data are presented as means ± SEM unless otherwise specified. Within each group there is an estimate of variation, and the variance between groups is similar. The number of replicates for each experiment and specific details of statistical analyses conducted were described in the figure legends or main text. Statistical significance between two groups was determined by two-tailed Student’s *t* test unless otherwise specified. All the statistical tests were justified for every figure and the data meet the assumptions of the tests. *P* value <0.05 was considered to be statistically significant. Data point is excluded if it deviates from mean with more than three standard deviations. Investigators were not blinded to the group allocation during the experiment and when assessing the outcome in all experiments including animal experiments.

### Supplementary information

Original Data File

SUPPLEMENTAL MATERIAL

checklist

## Data Availability

The data generated in this study are available upon request from the corresponding author.
