# Supplementary material for: FBXO32-mediated degradation of PTEN promotes lung adenocarcinoma progression
Source: Cell Death Dis. 2024 Apr 20;15(4):282. doi: 10.1038/s41419-024-06635-4 (PMC11032391; doi:10.1038/s41419-024-06635-4)
Supplement: Supplementary file 1 — Original Data File [file 41419_2024_6635_MOESM1_ESM.pptx]

## Slide 1
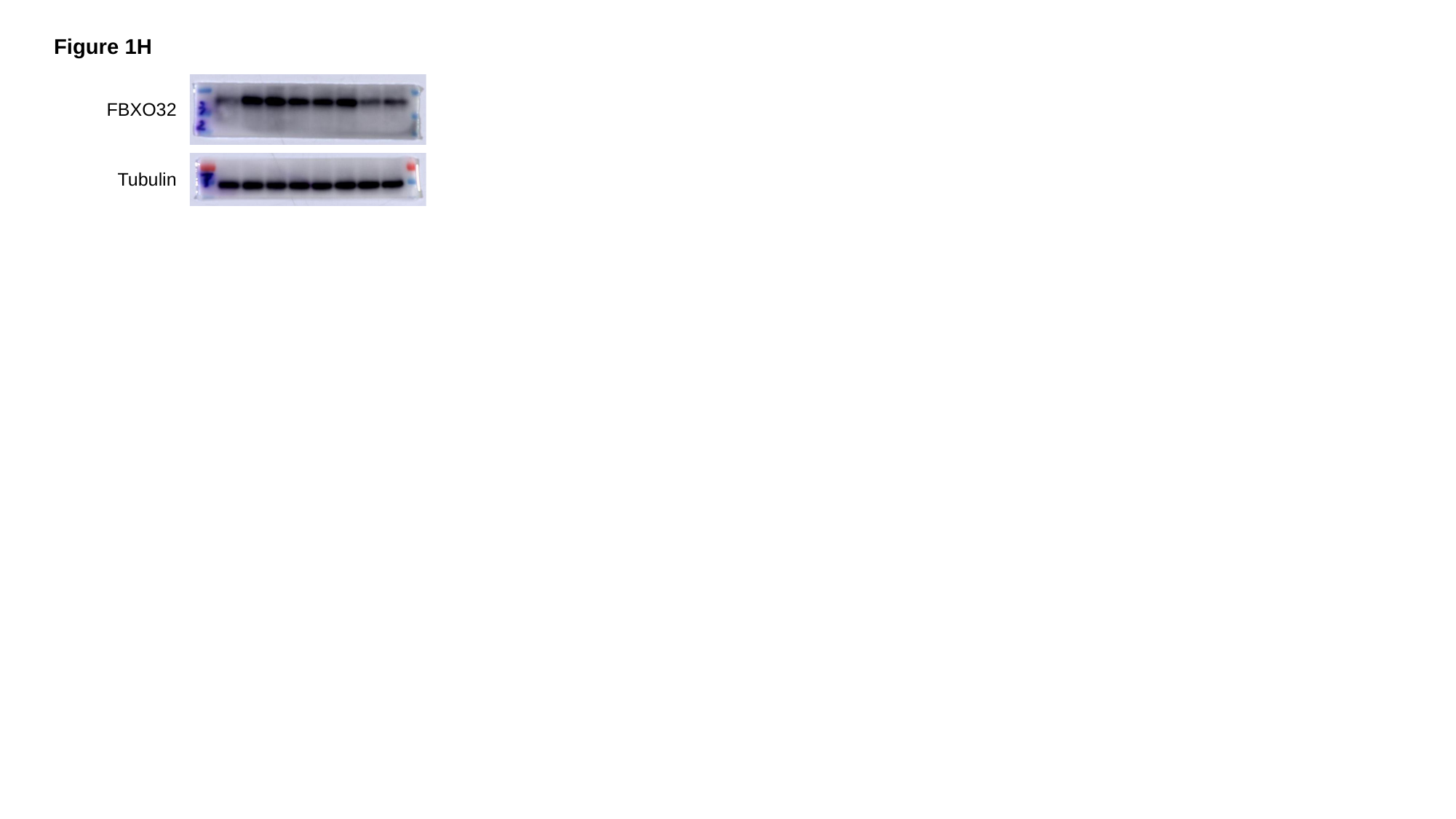

Figure 1H
FBXO32
Tubulin

## Slide 2
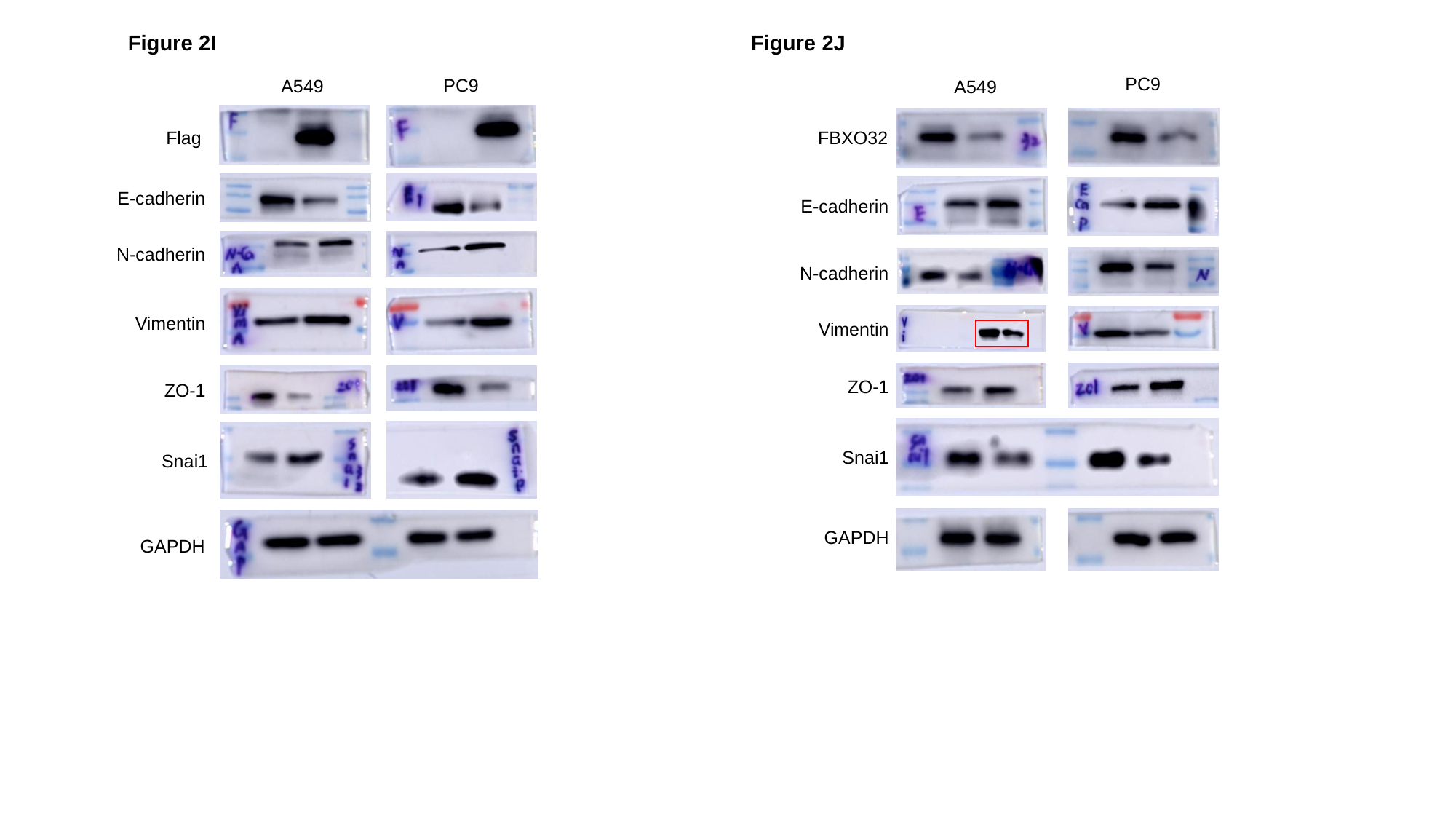

Figure 2I
Figure 2J
PC9
A549
E-cadherin
N-cadherin
Vimentin
ZO-1
Snai1
GAPDH
FBXO32
PC9
A549
E-cadherin
Vimentin
ZO-1
Snai1
GAPDH
N-cadherin
Flag

## Slide 3
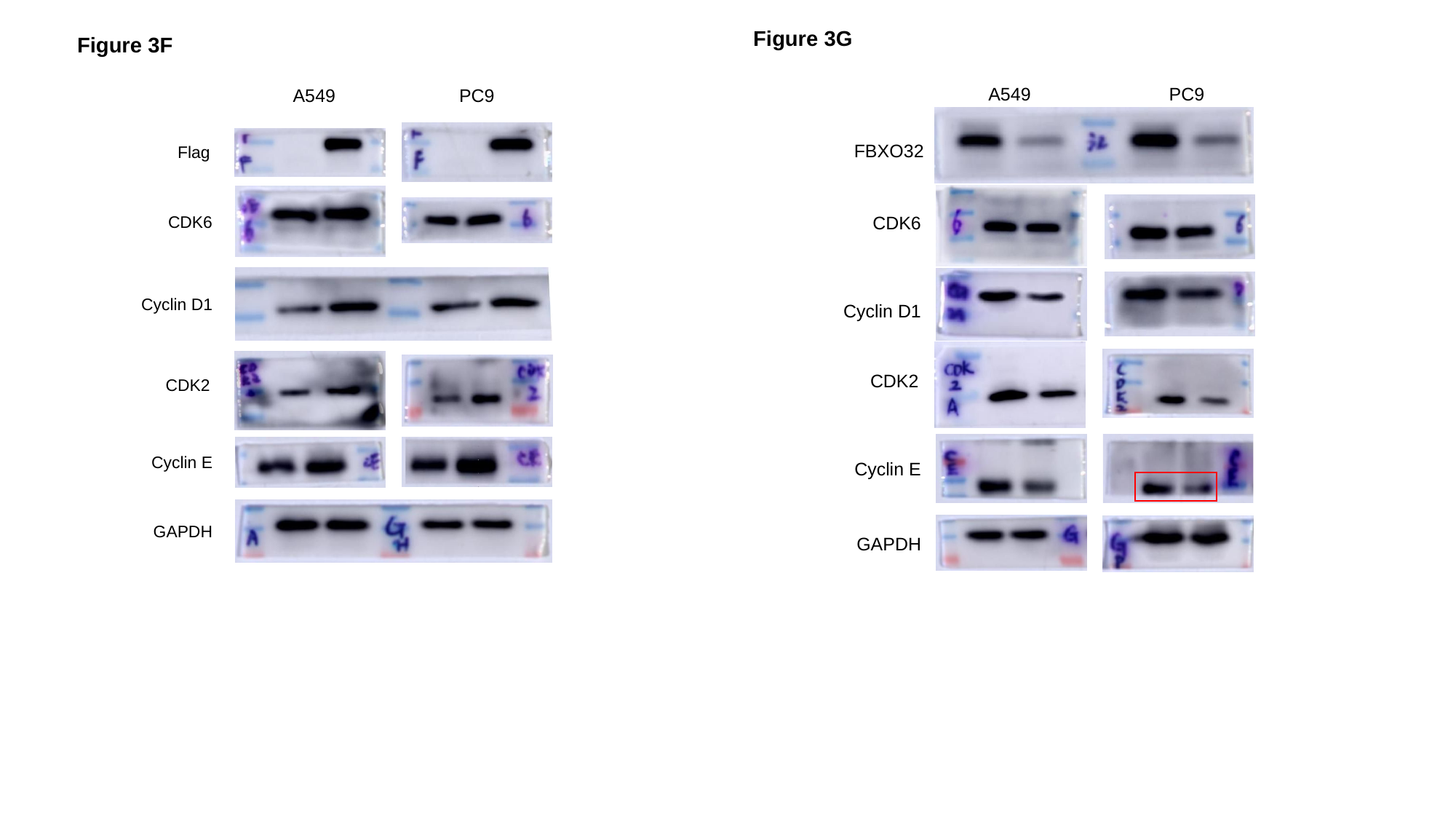

Figure 3G
Figure 3F
A549
PC9
CDK6
Cyclin D1
CDK2
Cyclin E
GAPDH
FBXO32
A549
PC9
CDK6
Cyclin D1
CDK2
Cyclin E
GAPDH
Flag

## Slide 4
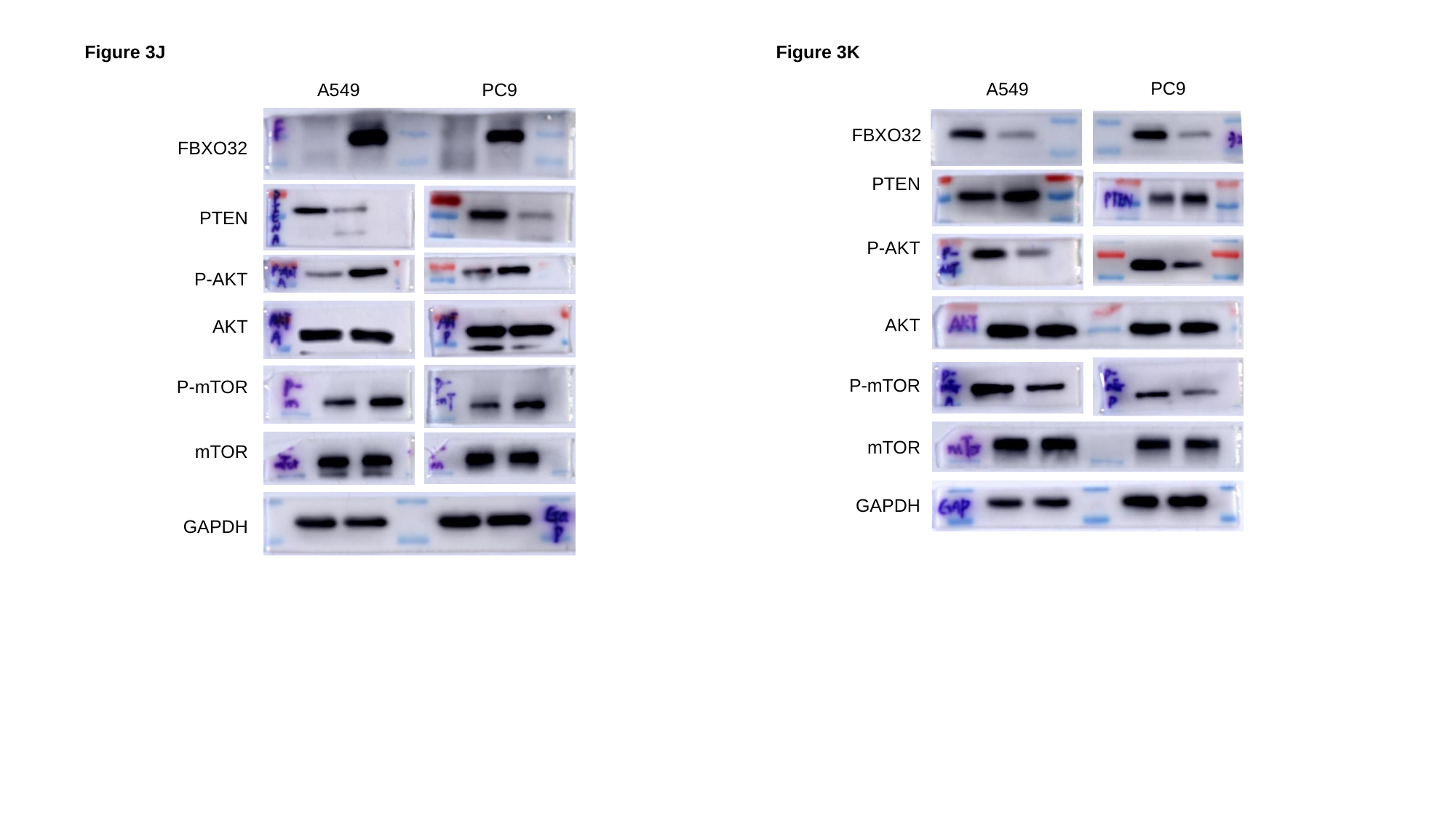

Figure 3J
Figure 3K
PC9
A549
PTEN
P-AKT
AKT
P-mTOR
mTOR
GAPDH
FBXO32
A549
PC9
PTEN
P-AKT
AKT
P-mTOR
mTOR
GAPDH
FBXO32

## Slide 5
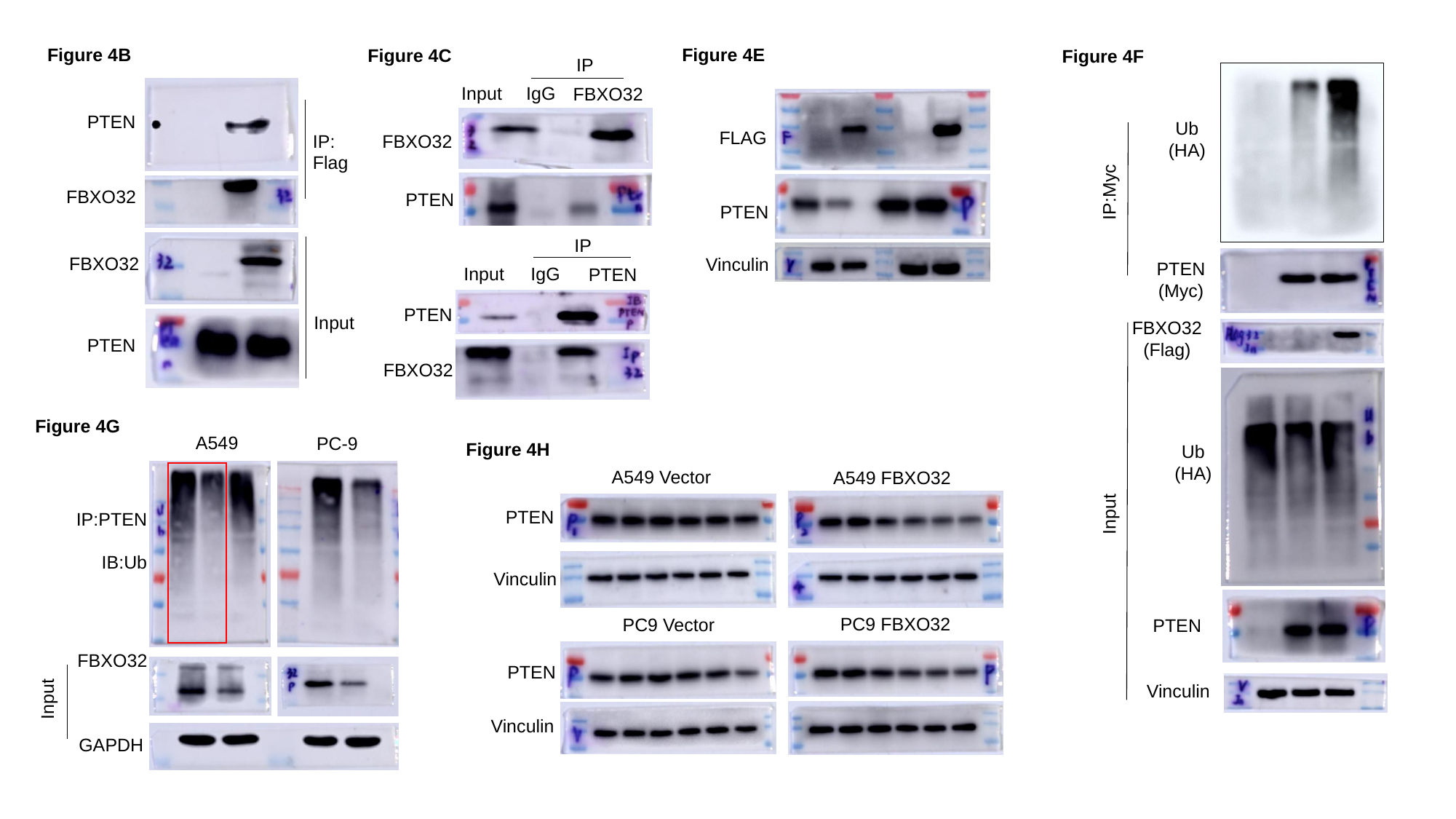

Figure 4E
Figure 4B
Figure 4C
Figure 4F
IP
Input
IgG
FBXO32
FBXO32
PTEN
Ub
(HA)
IP:Myc
PTEN
(Myc)
FBXO32
(Flag)
Ub
(HA)
Input
PTEN
Vinculin
PTEN
IP:
Flag
FBXO32
FBXO32
Input
PTEN
FLAG
PTEN
Vinculin
IP
Input
IgG
PTEN
PTEN
FBXO32
Figure 4G
A549
PC-9
IP:PTEN
IB:Ub
FBXO32
Input
GAPDH
Figure 4H
A549 Vector
A549 FBXO32
PTEN
Vinculin
PC9 FBXO32
PC9 Vector
PTEN
Vinculin

## Slide 6
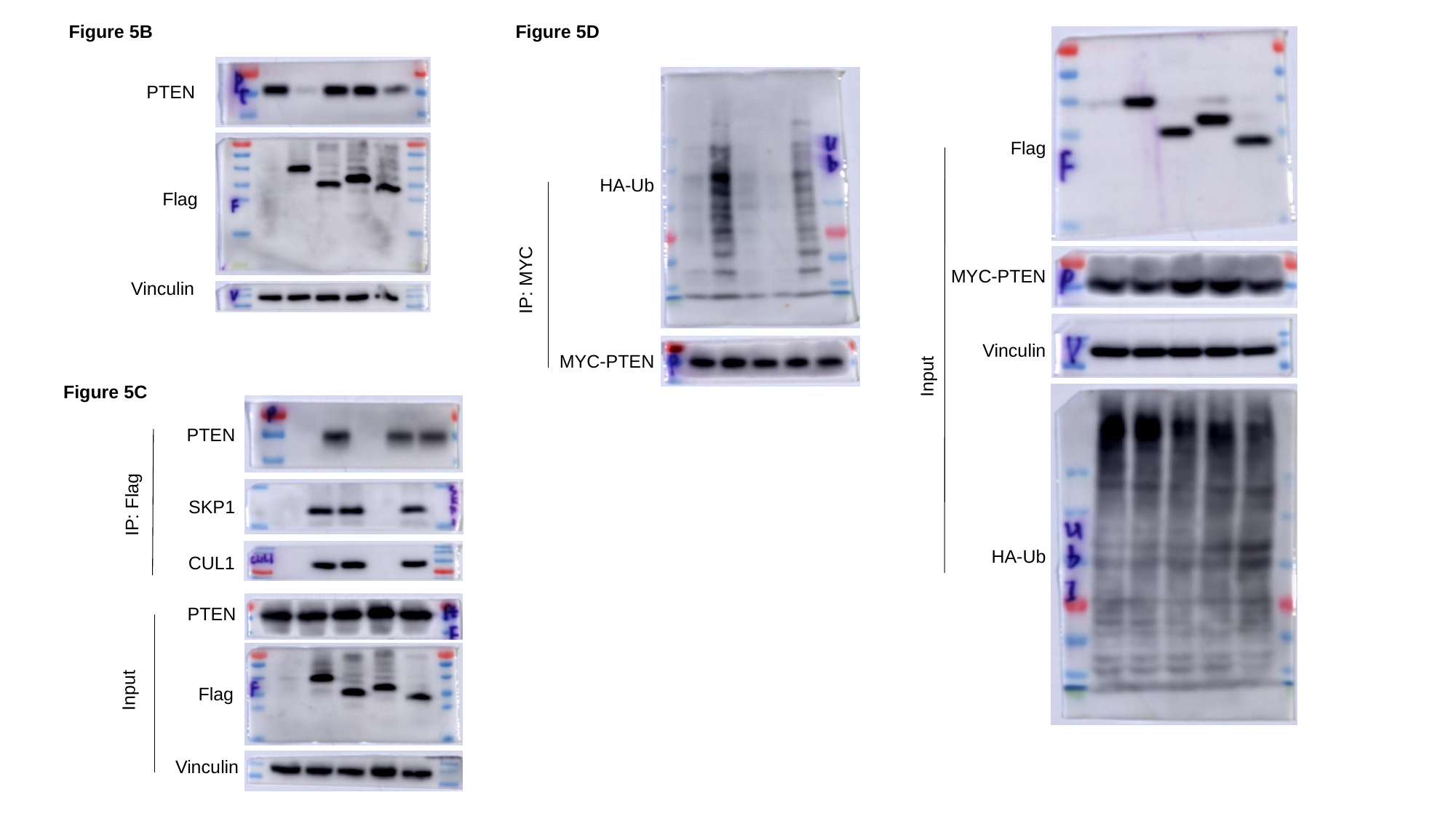

Figure 5B
Figure 5D
Flag
MYC-PTEN
Vinculin
Input
HA-Ub
HA-Ub
IP: MYC
MYC-PTEN
PTEN
Flag
Vinculin
PTEN
SKP1
IP: Flag
CUL1
PTEN
Input
Flag
Vinculin
Figure 5C

## Slide 7
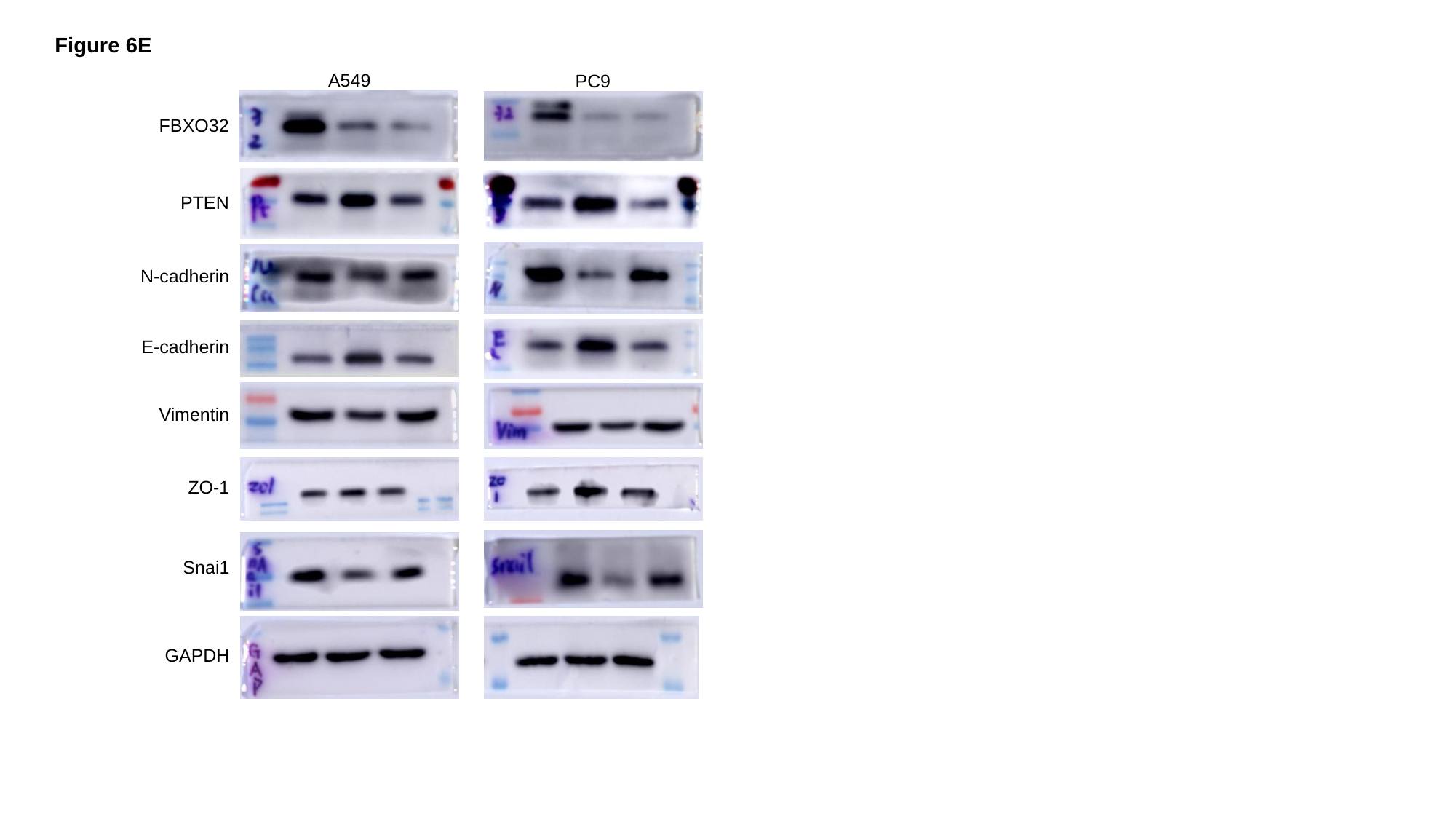

Figure 6E
A549
PC9
N-cadherin
E-cadherin
Vimentin
ZO-1
Snai1
GAPDH
FBXO32
PTEN

## Slide 8
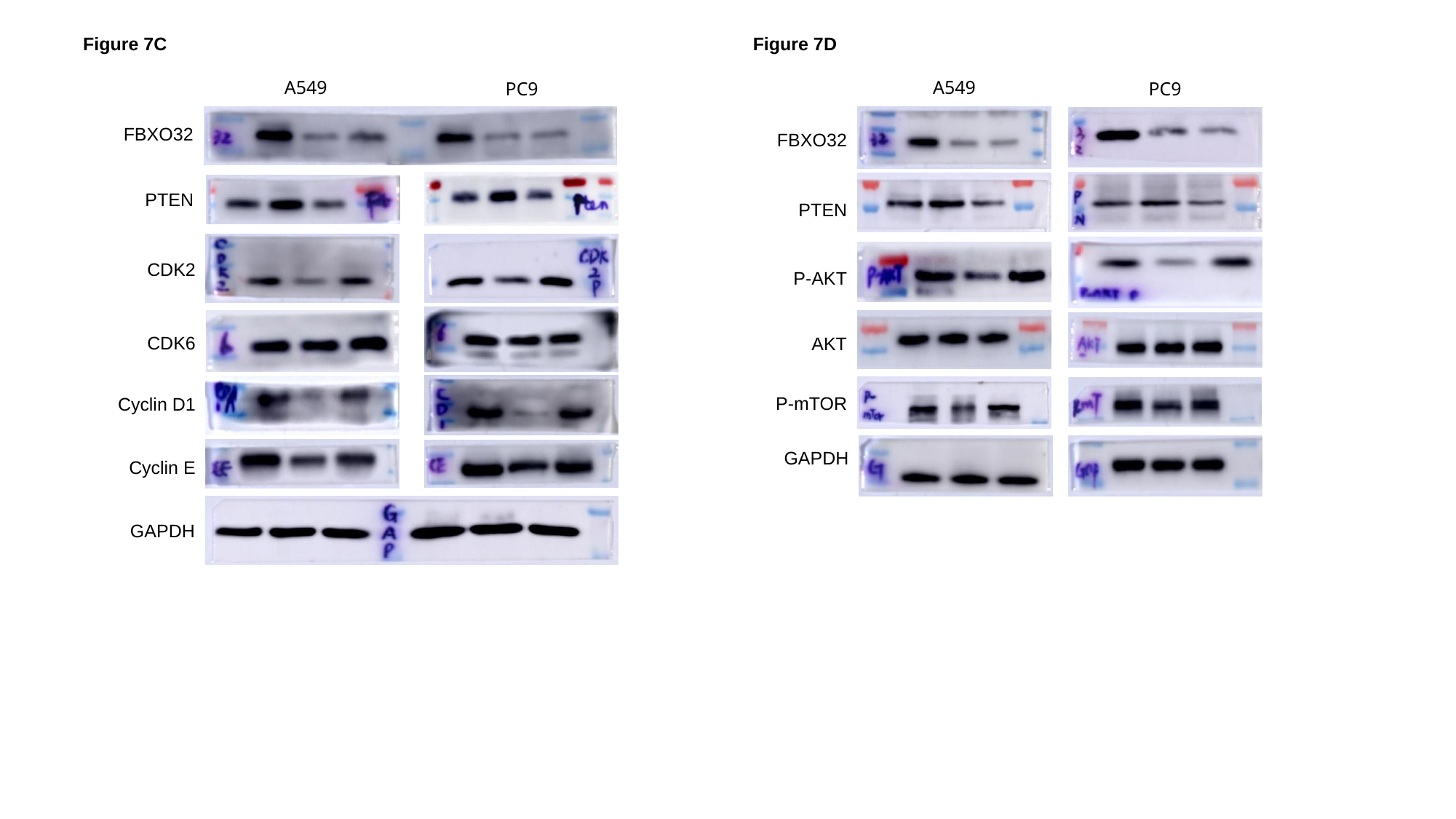

Figure 7C
Figure 7D
A549
PC9
FBXO32
PTEN
P-AKT
AKT
P-mTOR
GAPDH
A549
PC9
CDK2
CDK6
Cyclin D1
Cyclin E
GAPDH
FBXO32
PTEN
