# Supplementary material for: FBXO32-mediated degradation of PTEN promotes lung adenocarcinoma progression
Source: Cell Death Dis. 2024 Apr 20;15(4):282. doi: 10.1038/s41419-024-06635-4 (PMC11032391; doi:10.1038/s41419-024-06635-4)
Supplement: Supplementary file 2 — SUPPLEMENTAL MATERIAL [file 41419_2024_6635_MOESM2_ESM.docx]

**Supplementary Figures and Tables**


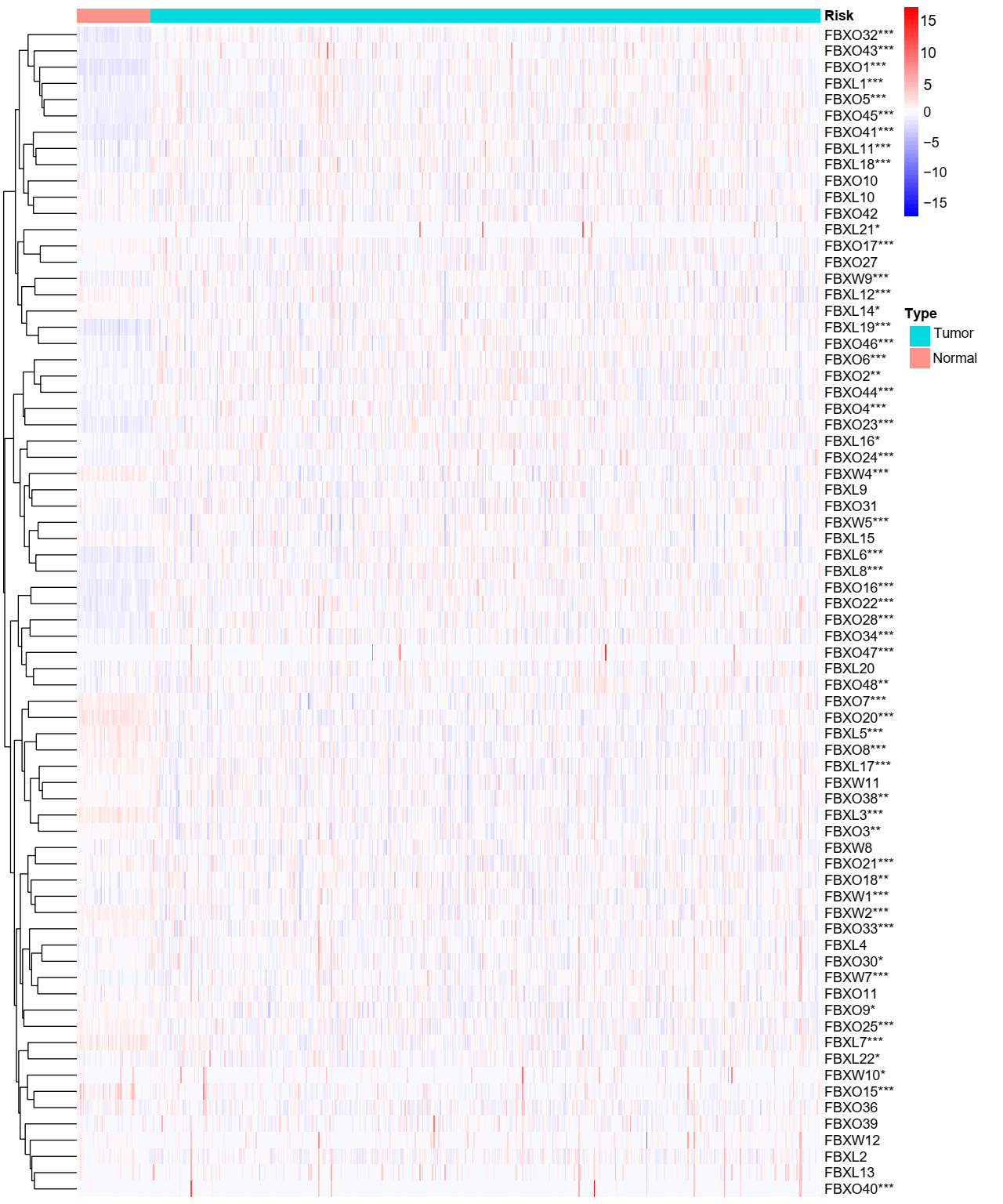


**Supplementary Figure 1** Heatmap showing the expression profiles of the F-box gene family in normal and LUAD (LUAD) samples from the TCGA database. **P* < 0.05, ** *P* < 0.01, *** *P* < 0.001. *P* values were determined by t-tests.


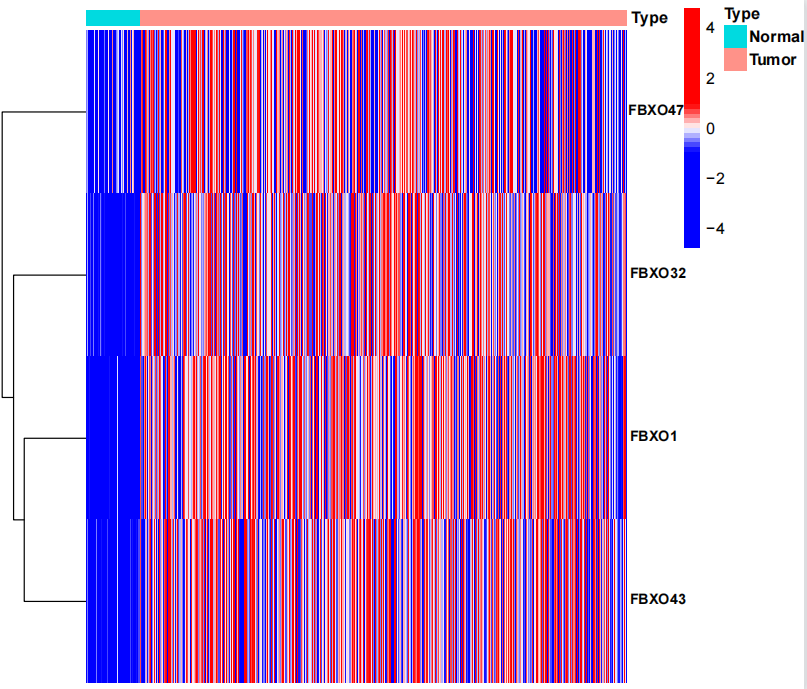


**Supplementary Figure 2** Heatmap showing the expression of FBXO47, FBXO32, FBXO1 and FBXO43 in normal lung tissues and LUAD tissues from the TCGA database.


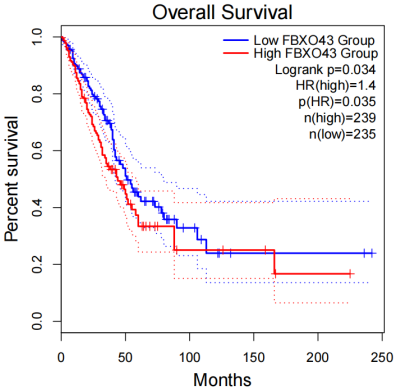

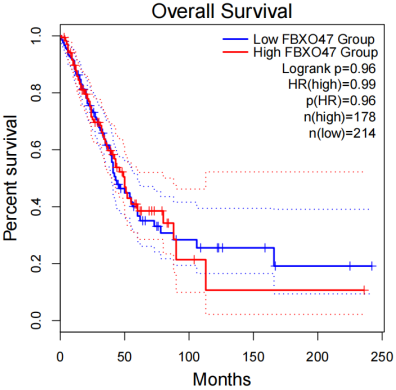


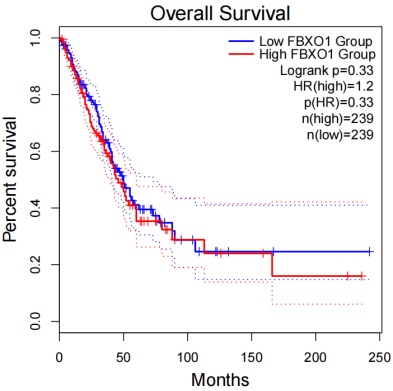


**Supplementary Figure 3** The prognostic value of FBXO1, FBXO47 and FBXO43 expression level in LUAD from the GEPIA database.


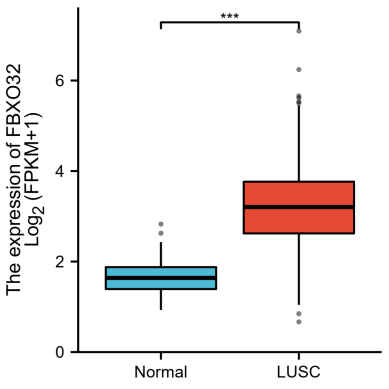

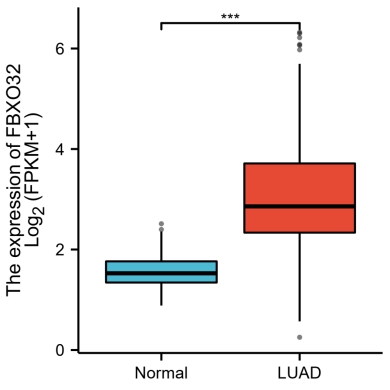

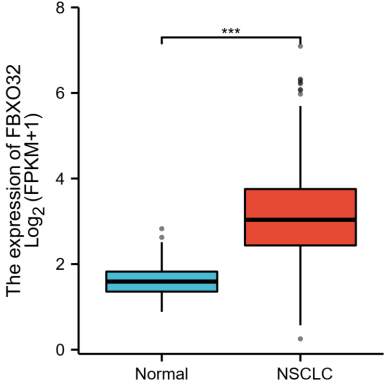


**Supplementary Figure 4** Boxplots showing differential expression of FBXO32 between normal lung samples and NSCLC (LUAD and LUSC) samples from TCGA database. ****P* < 0.001. *P* values were determined by t-tests.

**Supplementary Figure 5** The prognostic value of the FBXO32 expression level in LUSC from the GEPIA database.


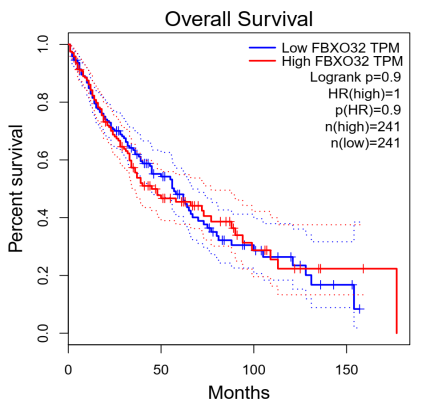


**LUSC**

**
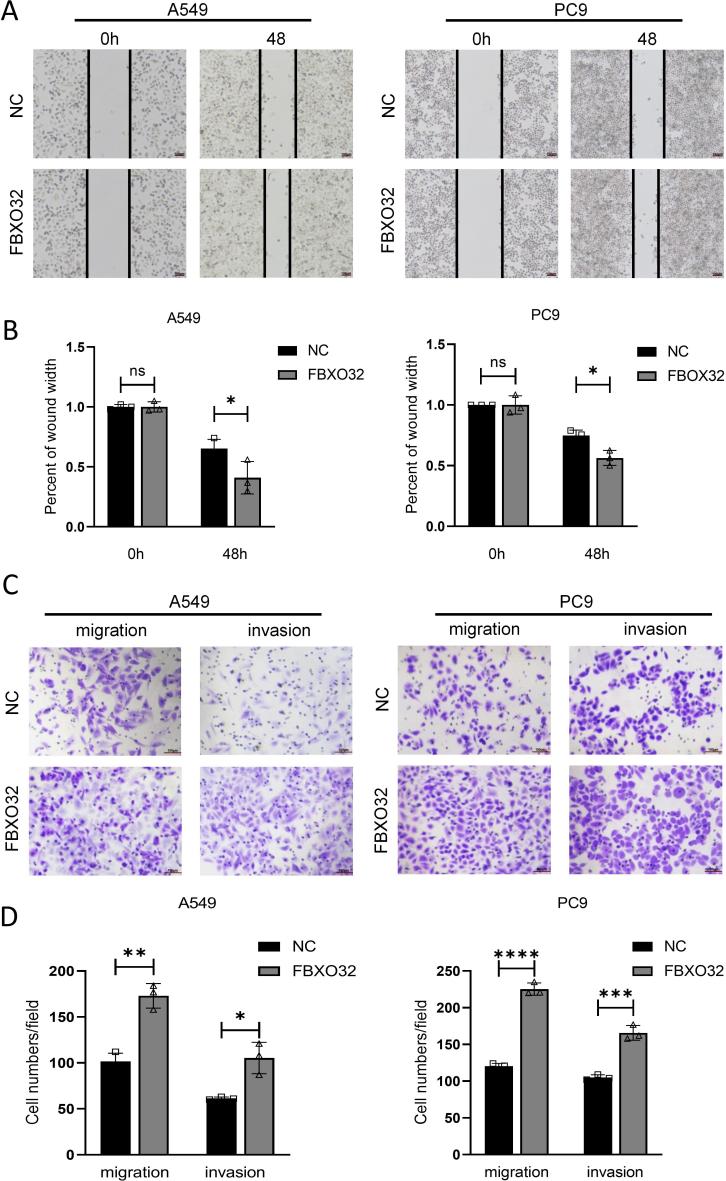
**

**Supplementary Figure 6** A-D Transwell assays and wound healing assays in A549 and PC9 cells after FBXO32 overexpression. Percent of wound width were measured and analyzed at 0h and 48h after the wound formation (B). Migration and invasion cells per field were counted and analyzed (D). **P* < 0.05; ***P* < 0.01; ns: no significance. *P* values were determined by Two-way ANOVA analysis. NC: E3 ligase deficient FBXO32 as negative control.


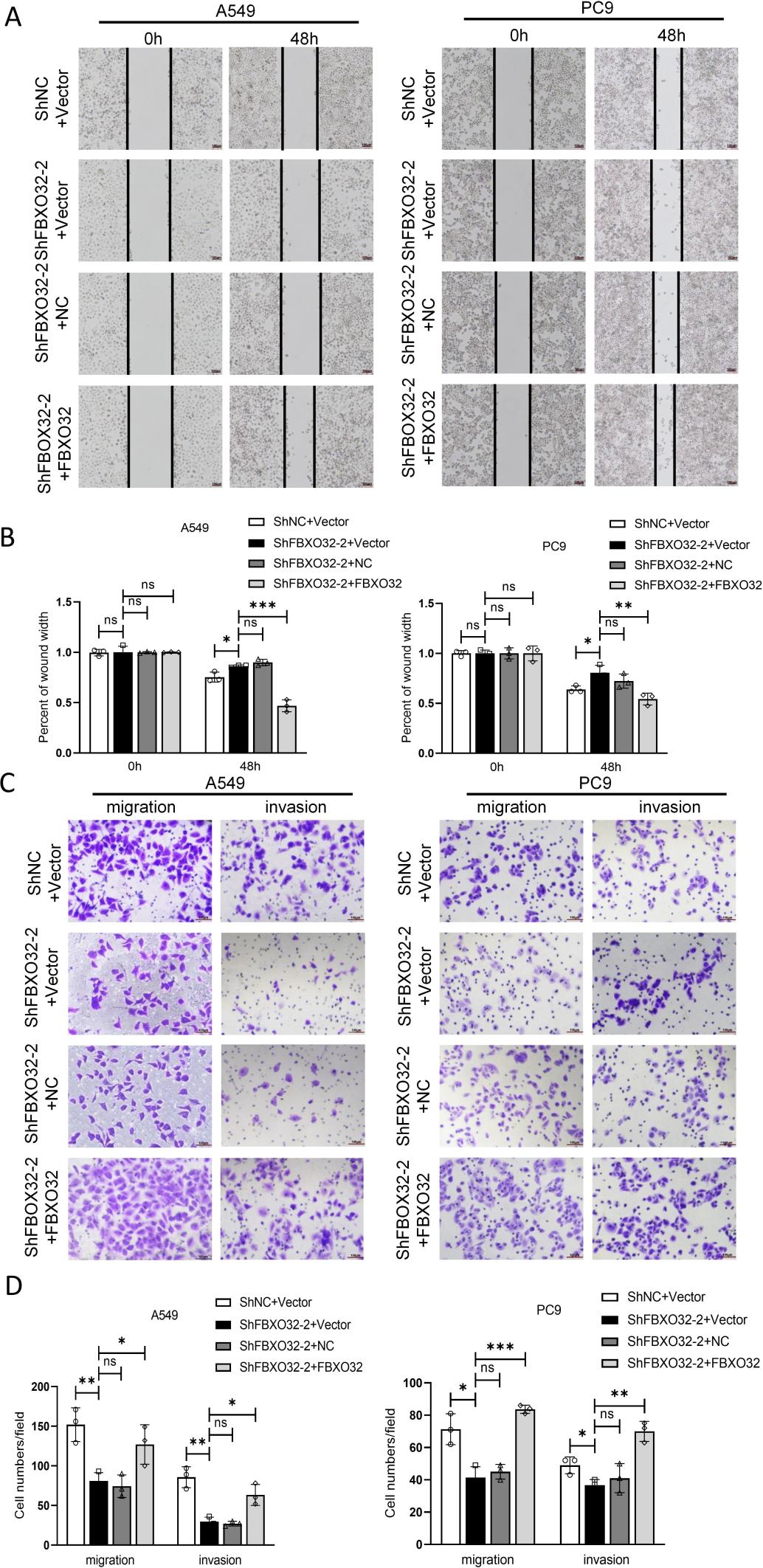


**Supplementary Figure 7** The ability of cancer cells to migrate and invade was assessed using the wound healing assay (A, B) and the transwell assay(C, D). Percent of wound width were measured at 0h and 48h after the wound formation (B). Migration and invasion cells per field were counted (D). **P* < 0.05; ***P* < 0.01; ****P* < 0.001; ns: no significance. *P* values were determined by Two-way ANOVA analysis. NC: E3 ligase deficient FBXO32 as negative control.


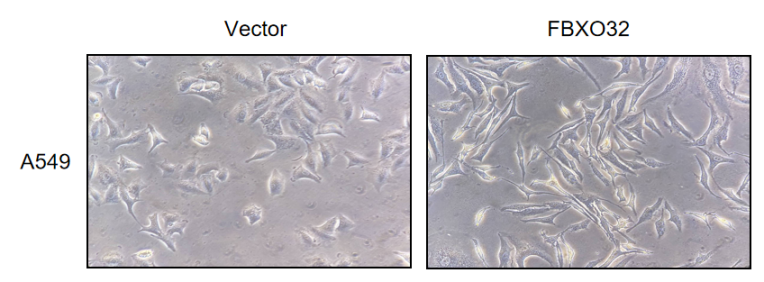


**Supplementary Figure 8** Images of the morphologic changes in A549 cells after transfection with the FBXO32 expression plasmid.


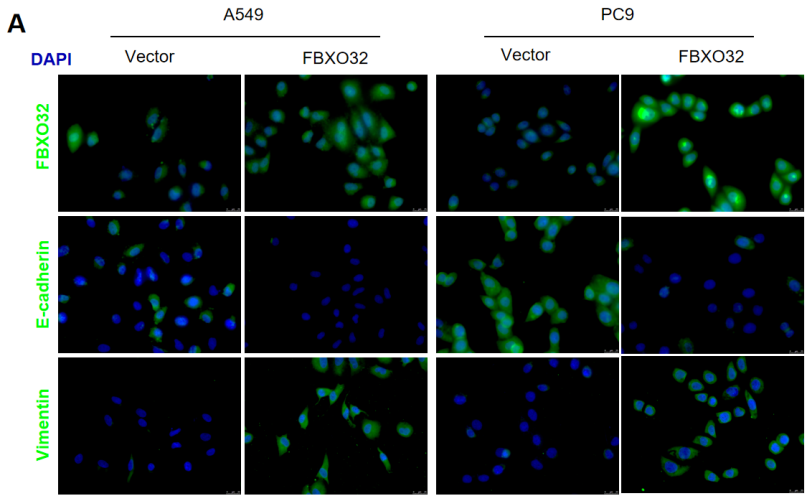


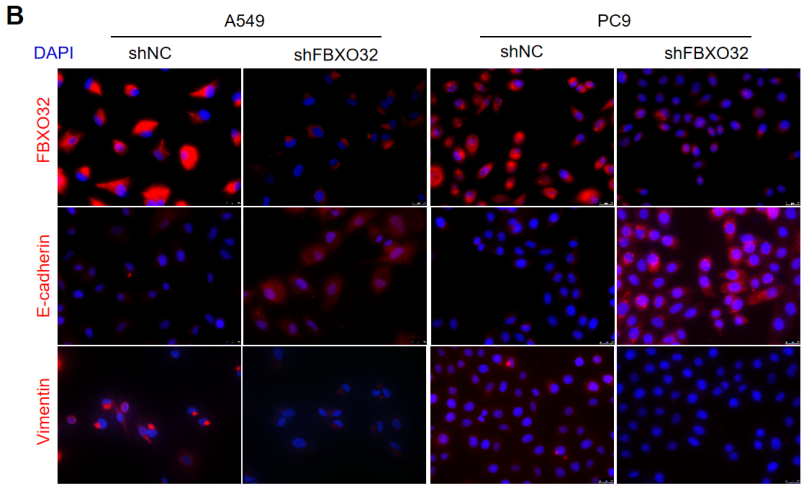


**Supplementary Figure 9** Images of immunofluorescent staining displaying the expression of FBXO32, E-Ca and Vimentin in A549 and PC9 cells after FBXO32 overexpression (A) or knockdown (B). Nuclear DNA was counterstained with DAPI (blue).


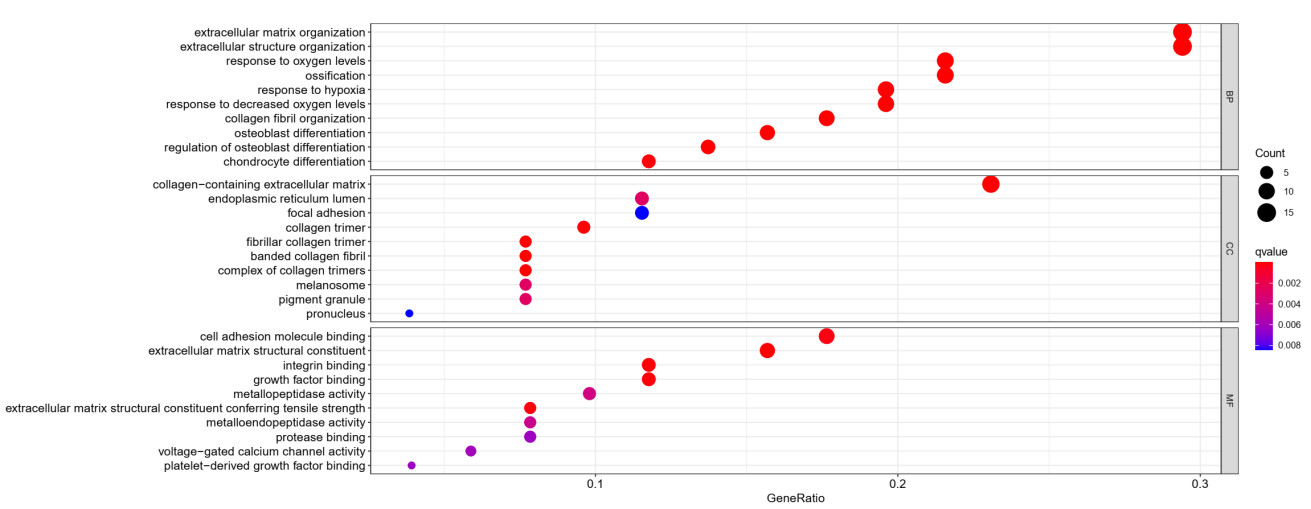


**Supplementary Figure 10** GO enrichment analysis of FBXO32 in LUAD with data from the TCGA database.


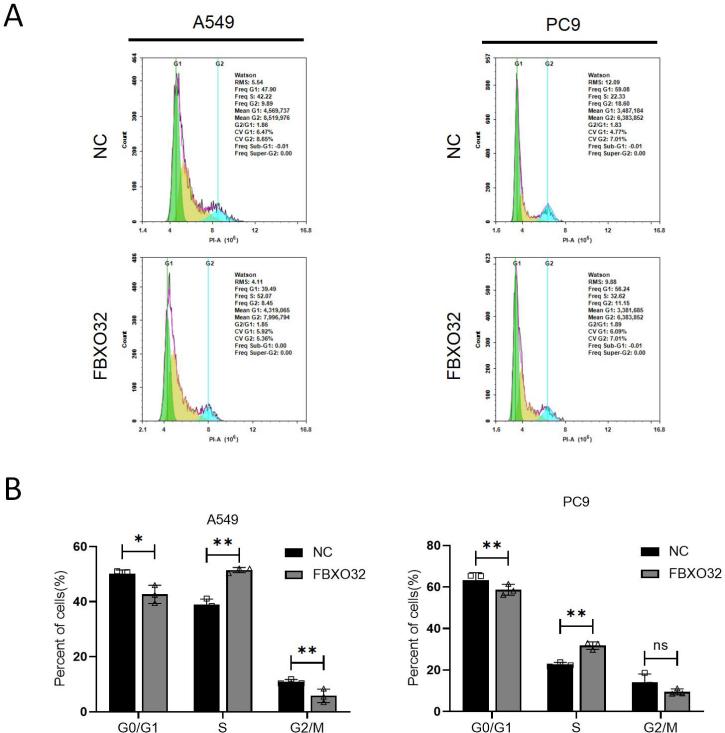


**Supplementary Figure 11** FACS analysis showing cell cycle progression in A549 and PC9 cells after FBXO32 overexpression (A). Percentage of G0/G1 phase, S phase or G2/M phase cells were analyzed (B). **P* < 0.05; ***P* < 0.01; *** *P* < 0.001; ns: not significant. *P* values were determined by Two-way ANOVA analysis. NC: E3 ligase deficient FBXO32 as negative control.


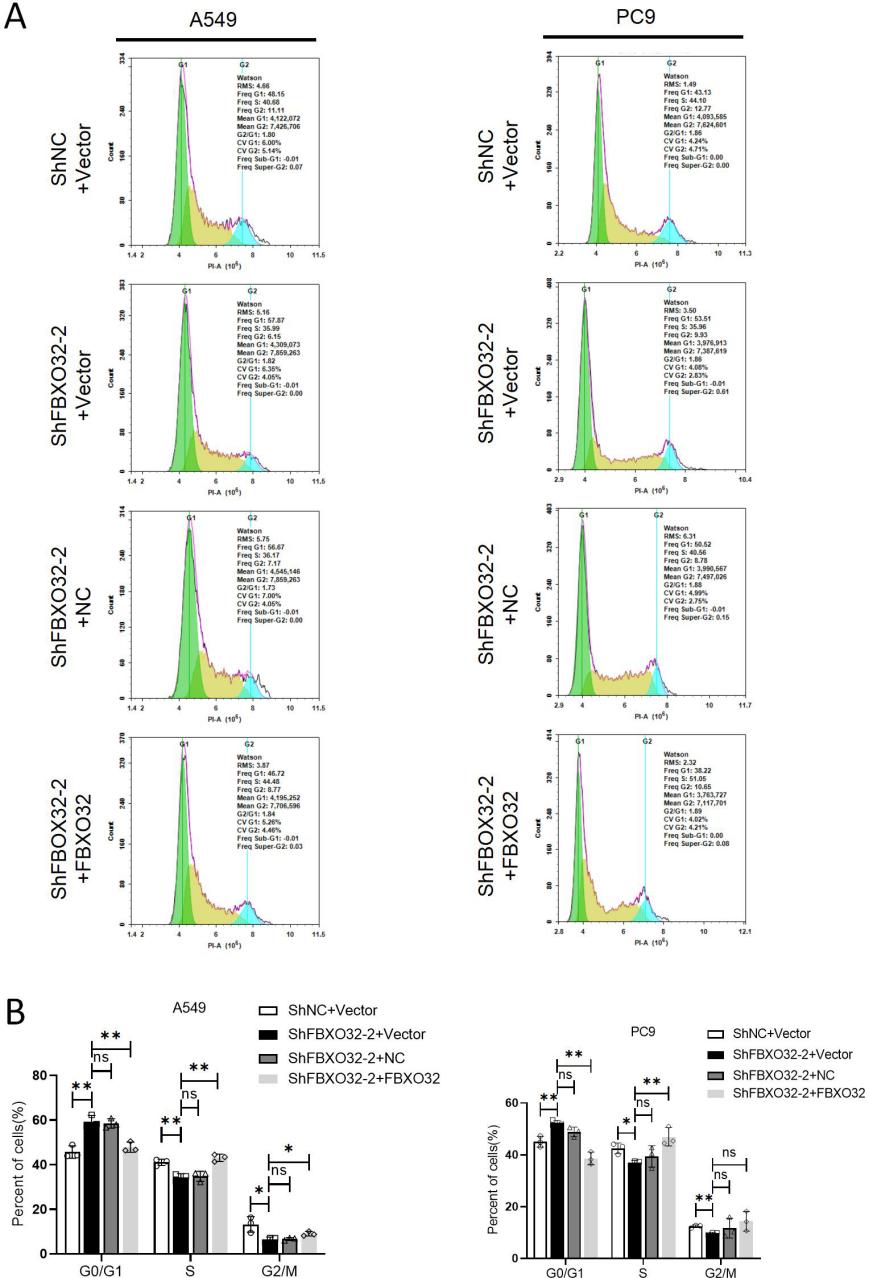


**Supplementary Figure 12** Cell cycle progression was analyzed by FACS (A). Percentage of G0/G1 phase, S phase or G2/M phase cells were analyzed (B). **P* < 0.05; ***P* < 0.01; ns: not significant. *P* values were determined by Two-way ANOVA analysis. NC: E3 ligase deficient FBXO32 as negative control.


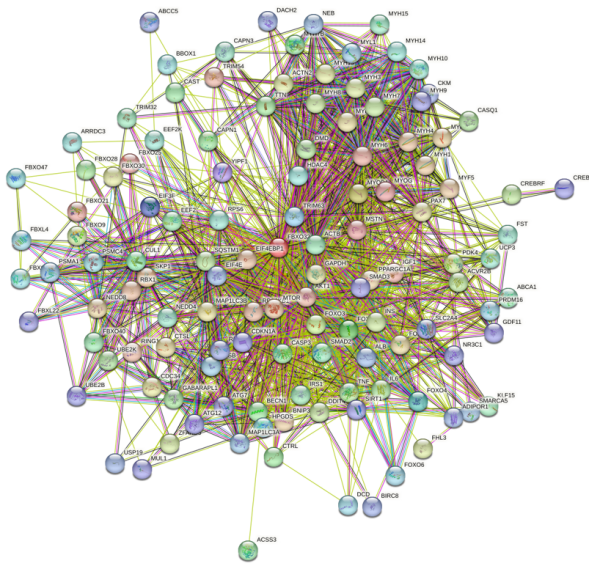


**Supplementary Figure 13** The protein interaction network of FBXO32 analyzed by STRING database.


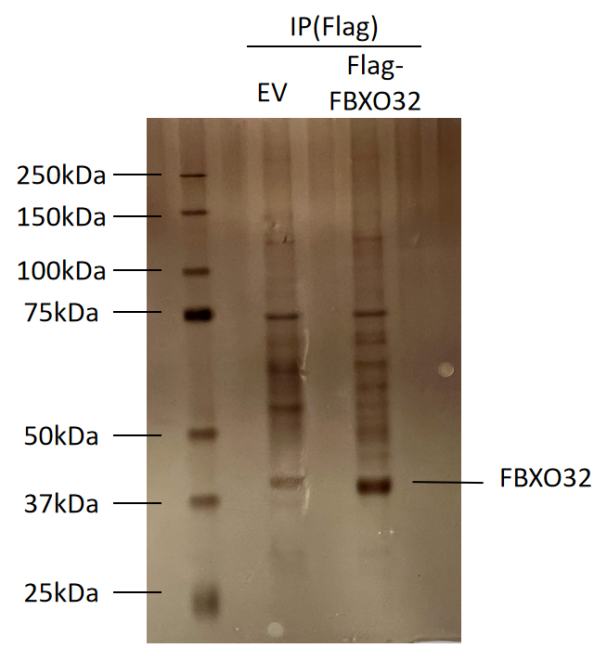


**Supplementary Figure 14** Sliver staining image of SDS-polyacrylamide gel.


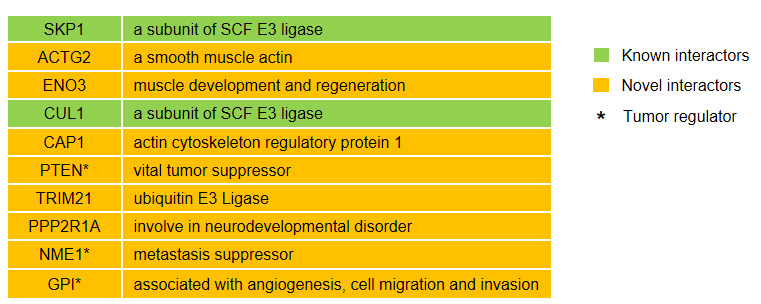


**Supplementary Figure 15** Top 10 interactors of FBXO32 analyzed by MS.


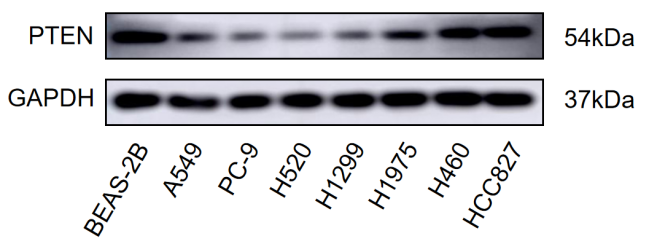


**Supplementary Figure 16** PTEN expression in normal lung epithelial cells (BEAS-2B) and lung cancer cells (A549/PC9/H520/H1299/H1975/H460/HCC827) were measured by Western blotting.


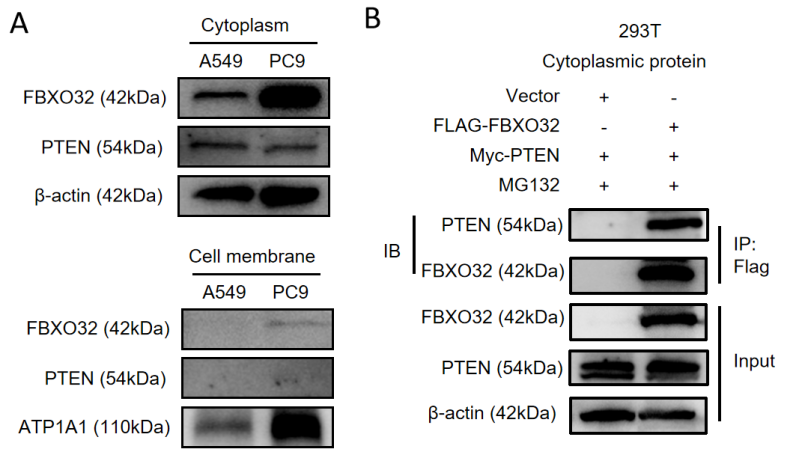


**Supplementary Figure 17** The expression of FBXO32 and PTEN in cytoplasm and cell membrane of LUAD cell lines (A). Exogenous coimmunoprecipitation (co-IP) experiments on cytoplasmic proteins of 293T cells to detect the interaction between FBXO32 and PTEN (B).


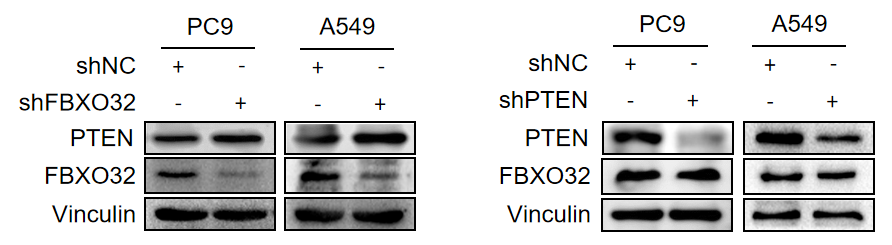


**Supplementary Figure 18** The expression of PTEN after FBXO32 knockdown and the expression of FBXO32 after PTEN knockdown in A549 and PC9 cell lines as measured by Western blotting.


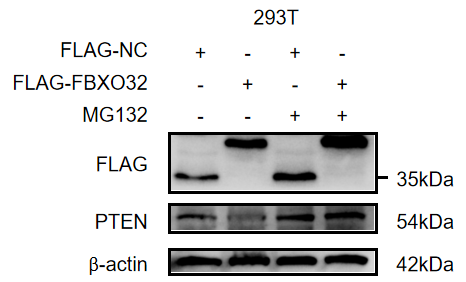


**Supplementary Figure 19** Expression of PTEN in transfected 293T cells with or without proteasome inhibitor MG132 treatment for 6 h as measured by Western blotting. NC represent E3 ligase deficient FBXO32.


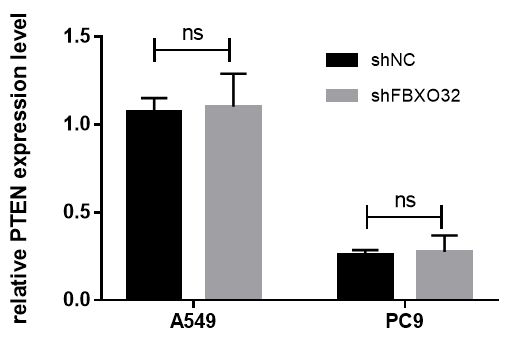


**Supplementary Figure 20** The relative mRNA expression of PTEN after FBXO32 knockdown as measured by RT-qPCR.


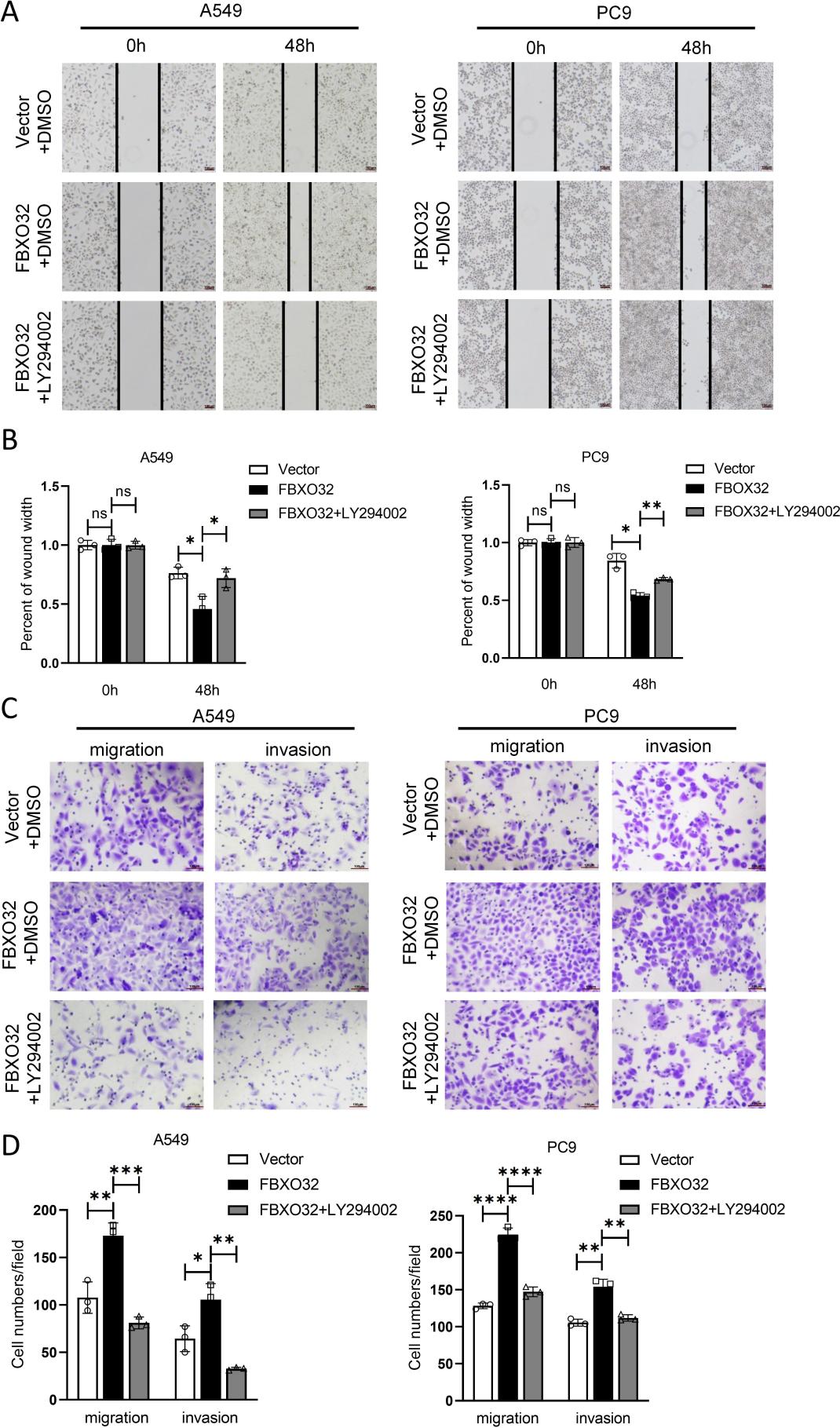


**Supplementary Figure 21** A-D Transwell assays and wound healing assays in A549 and PC9 cells with PI3k inhibitor LY294002 after FBXO32 overexpression. Percent of wound width were measured and analyzed at 0h and 48h after the wound formation (B). Migration and invasion cells per field were counted and analyzed (D). **P* < 0.05; ***P* < 0.01; ****P* < 0.001; ns: no significance. *P* values were determined by Two-way ANOVA analysis.

| Supplementary table 1 The expression of FBXO32 in LUAD and normal lung tissues. | | | | | |
| --- | --- | --- | --- | --- | --- |
|  | FBXO32 |  |  | χ2 | P value |
|  | Low | High | Total |  |  |
| Normal | 43 | 25 | 68 | 14.683 | <0.001 |
| Tumor | 38 | 74 | 112 |  |  |

**Supplementary table 2** Univariate Cox regression analysis of overall survival in LUAD patients

|  | B | SE | Wald | Sig. | Exp(B) | 95.0% CI for Exp(B) | |
| --- | --- | --- | --- | --- | --- | --- | --- |
|  |  |  |  |  |  | Lower | Upper |
| Gender | -0.144 | 0.275 | 0.273 | 0.601 | 0.866 | 0.505 | 1.485 |
| Smoking | -0.158 | 0.277 | 0.323 | 0.570 | 0.854 | 0.496 | 1.471 |
| Age | -0.221 | 0.287 | 0.589 | 0.443 | 0.802 | 0.457 | 1.409 |
| Differentiation | 0.311 | 0.207 | 2.249 | 0.134 | 1.364 | 0.909 | 2.048 |
| Tumor | 0.615 | 0.210 | 8.586 | 0.003 | 1.850 | 1.226 | 2.792 |
| LN metastasis | 1.023 | 0.168 | 37.020 | 0.000 | 2.782 | 2.001 | 3.868 |
| TNM staging | 1.146 | 0.191 | 36.025 | 0.000 | 3.145 | 2.164 | 4.573 |
| Lobe | -0.105 | 0.279 | 0.141 | 0.708 | 0.901 | 0.521 | 1.556 |
| Loaction | -0.497 | 0.329 | 2.290 | 0.130 | 0.608 | 0.319 | 1.158 |
| FBXO32 | 0.807 | 0.321 | 6.316 | 0.012 | 2.242 | 1.195 | 4.209 |

**Supplementary table 3** Multivariate Cox regression analysis of overall survival in LUAD patients

|  | B | SE | Wald | Sig. | Exp(B) | 95.0% CI for Exp(B) | |
| --- | --- | --- | --- | --- | --- | --- | --- |
|  |  |  |  |  |  | Lower | Upper |
| Tumor | 0.574 | 0.222 | 6.661 | 0.010 | 1.775 | 1.148 | 2.745 |
| LN metastasis | 0.924 | 0.166 | 30.862 | 0.000 | 2.519 | 1.819 | 3.49 |
| FBXO32 | 0.724 | 0.331 | 4.787 | 0.029 | 2.063 | 1.078 | 3.945 |

| **Supplementary table 4**  Correlation of FBXO32 and PTEN expression in LUAD. | | | | |
| --- | --- | --- | --- | --- |
|  | FBXO32 |  | r | *P* value |
|  | Low | High |  |  |
| PTEN |  |  |  |  |
| Low | 12 | 55 | -0.413 | <0.001 |
| High | 26 | 19 |  |  |

| **Supplementary table 5**. The target sequences of lentiviral shRNAs used in this study. | |
| --- | --- |
| Lentiviral shRNA | Target sequence |
| shNC | 5′-TTCTCCGAACGTGTCACGT-3′ |
| shFBXO32 | 5'-CAACAAGGAGGTATACAAT-3' |
| shFBXO32-2 | 5'-GGAAGAAGATGTATTTCAAAC-3' |
| shPTEN | 5'-GCGCTATGTGTATTATTAT-3' |

| **Supplementary table 6**. RT-PCR primers used in this study. | |
| --- | --- |
| Primer name | Squence |
| β‐actin F | 5′‐GCTCGTCGTCGACAACGGCTC‐3′ |
| β‐actin R | 5′‐CAAACATGATCTGGGTCATCTTCTC‐3ʹ |
| FBXO32 F | 5’- AGTGAGGACCGGCTACTGTG -3’ |
| FBXO32 R | 5’- GATCAAACGCTTGCGAATCT -3’ |
| PTEN F | 5′- GGACGAACTGGTGTAATGAT-3′ |
| PTEN R | 5′- GGACGAACTGGTGTAATGAT-3′ |

| **Supplementary table 7**. List of online databases used in this study. | |
| --- | --- |
| Database | Online link |
| Genomic Data Commons | <https://portal.gdc.cancer.gov/> |
| Kaplan-Meier Plotter database | <http://kmplot.com/analysis/> |
| GEPIA database | <http://gepia.cancer-pku.cn/> |
| JASPAR database | [http://jaspar.genereg.net](http://jaspar.genereg.net/) |
| TIMER database | <https://cistrome.shinyapps.io/timer/> |
| Gene set enrichment analysis (GSEA) | <https://www.broadlnstitute.org/gsea/> |
| STRING database | <https://string-db.org/> |
| DAVID database | <https://david.ncifcrf.gov/> |
